# Controlling transmembrane ion transport *via* photo-regulated carrier mobility†

**DOI:** 10.1039/d2sc03322d

**Published:** 2022-07-08

**Authors:** Laura E. Bickerton, Matthew J. Langton

**Affiliations:** Chemistry Research Laboratory, Department of Chemistry, University of Oxford 12 Mansfield Road Oxford OX1 3TA UK matthew.langton@chem.ox.ac.uk

## Abstract

Stimuli-responsive transmembrane ion carriers allow for targeted and controllable transport activity, with potential applications as therapeutics for channelopathies and cancer, and in fundamental studies into ion transport phenomena. These applications require OFF–ON activation from a fully inactive state which does not exhibit background activity, but this remains challenging to achieve with synthetic transport systems. Here we introduce a novel mechanism for photo-gating mobile ion carriers, which involves modulating the mobility of the carriers within the lipid bilayer membrane. By appending a membrane-targeting anchor to the carrier using a photo-cleavable linker, the carrier's ion transport activity is fully switched off by suppressing its ability to shuttle between the two aqueous-membrane interfaces of the bilayer. The system can be reactivated rapidly *in situ* within the membrane by photo-triggered cleavage of the anchor to release the mobile ion carrier. This approach does not involve direct functionalization of the ion binding site of the carrier, and so does not require the *de novo* design of novel ion binding motifs to implement the photo-caging of activity. This work demonstrates that controlling the mobility of artificial transport systems enables precise control over activity, opening up new avenues for spatio-temporally targeted ionophores.

## Abstract 2

Photo-gated anion transport is achieved by modulating the mobility of mobile carriers within a lipid bilayer membrane, using a photo-cleavable membrane anchor. This enables *in situ*, off–on activation of transport in vesicles.

## Graphical abstract

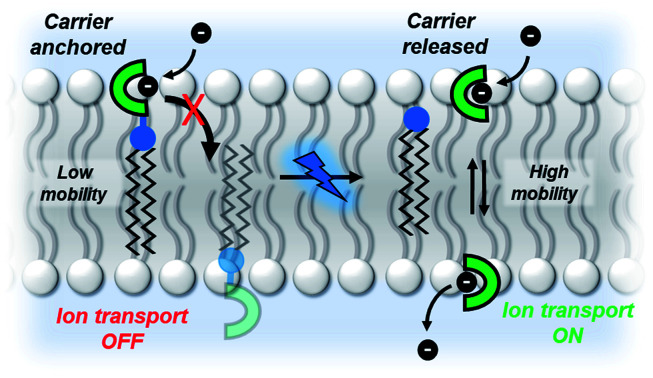

## Introduction

A key feature of transmembrane ion channels and pumps is their ability to respond to chemical and physical changes in their environment, including small molecule binding, membrane potential and light. The development of synthetic ion channels and mobile carriers has attracted significant interest, particularly for anions,^1–3^ both as fundamental tools for investigating ion transport processes, but also as potential therapeutics.^4–6^ Stimuli-responsive ion transporters have also begun to emerge, with the aim of achieving spatio-temporal control over transport.^7^ Examples of responsive ion channels include those triggered by light,^8^ membrane tension,^9^ potential^10^ and small molecule ligands.^11–16^ However, such systems remain underdeveloped, and readily accessible and versatile small molecule carrier analogues with responsive behavior are particularly rare. Furthermore, it remains highly challenging to design responsive transporters that can switch from a fully inactive OFF state, with no background transport activity, to an active ON state.

Recently, a number of different approaches to controlling the activity of mobile ion carriers have been reported.^7^ Photo-switchable transporters, based on a molecular photo-switch such as an azobenzene^17–20^ or stilbene^21^ with appended binding sites, allow for reversible activation by modulating the anion binding affinity. However, in these photo-switchable systems the OFF isomers typically have some background transport activity because they can inevitably bind anions to some extent. Azobenzene switching has also been used to regulate transport *via* a relay mechanism^22^ and to control a membrane-spanning [2]rotaxane.^23^ Irreversible redox modulation of anion binding affinity and therefore transport activity has also been reported for chalcogen bonding anionophores.^24,25^ Caged transporters, also referred to as pro-carriers, in which the anion binding site is blocked by a group that can be subsequently removed, also allow for OFF–ON activation. Such examples include a photo-caged indole-2-carboxamide anion receptor^26^ and a glutathione-responsive Au(iii) caged system.^27^ However both systems, whilst enabling effective control over ion transport, required *de novo* anionophore design with a binding site optimized for incorporation of the caging group. Finally, caging with photo- or redox-labile hydrophilic groups inhibits transport by preventing membrane uptake of the pro-carrier from the aqueous phase, until these groups are removed by a de-caging reaction.^28,29^ This approach allows for regulation of activity, but the hydrophilic pro-carriers are not associated to the membrane prior to activation, which is disadvantageous for spatially targeted activation applications. Stimuli-responsive caged anionophores with excellent OFF–ON activation profiles, which also target lipid membranes, are therefore highly attractive targets.

Herein, we introduce a new mechanism for precisely regulating anion transport, with complete binary OFF–ON activation behavior and membrane-targeting ability. The mechanism involves caging transporters through control over their mobility, by appending anchoring groups that inhibit the membrane translocation of the carrier that is necessary for transport. The anchor is connected to the carrier *via* a photo-cleavable linker, which enables the activity of the carrier to be switched ON by photo-triggered bond cleavage. This strategy also allows for the anion binding site to be preserved, unlike existing caging strategies that block the receptor and thus require *de novo* design of transporters with binding sites amenable to caging.^26,27^ This approach should therefore be widely applicable to caging existing transport systems, and we demonstrate the concept by converting both triazole and squaramide-based anion transporters into fully OFF–ON photo-caged systems.

## Photo-cleavable anchor design and synthesis

**Fig. 1:**
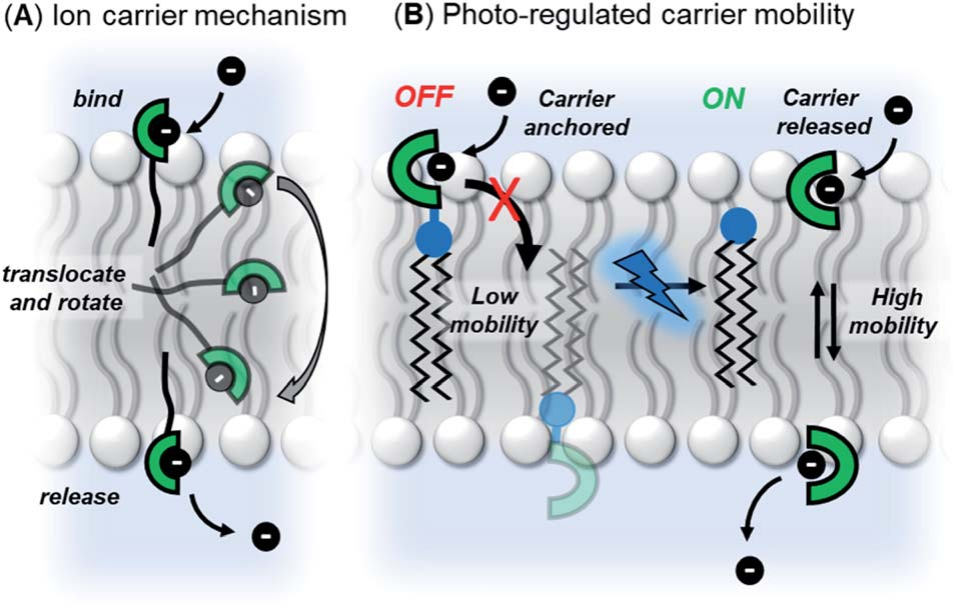
(A) Ion transport *via* a carrier mechanism. This requires translocation and rotation of the carrier to bind and release the ions at opposite interfaces of the membrane, which is inhibited by long pendant alkyl chains.^30^ (B) This work: Photo-regulated carrier mobility. The anchor (black) appended to the carrier (green) inhibits transport. A photo-cleavable linker (blue) enables the anchor to be removed with photo-irradiation, releasing the carrier and switching on transport.

Our strategy was to append a membrane anchoring motif to existing anion carrier designs, to inhibit their mobility within the bilayer and switch-off transport. It has previously been reported by Davis and co-workers that long alkyl chains appended to anion carriers decreases transport activity by slowing the rotation of the carrier–anion complex within the membrane *via* increased van der Waals interactions with the phospholipid tails.^30^ The transmembrane tumbling of the carrier is a critical step in the anion transport process; the receptor must approach the water-membrane interface to bind the anion, and then the anion–carrier complex itself must translocate across the membrane and rotate in order to deliver the anion to the opposite interface (Fig. 1A). The reduction of transport activity for carriers with long alkyl chains has also been observed for a number of other anion carrier systems.^31,32^ We therefore identified a dodecyl anchor as an appropriate motif to inhibit mobility, which has previously been used for membrane-targeting of fluorescent sensors.^33^ The general design of the caged transporter system is shown in Fig. 1B: the dodecyl membrane anchor is linked to the carrier by a photo-cleavable group, such that irradiation of the system with a suitable wavelength of light releases the carrier from the anchor and switches ON transport activity.

**Fig. 2:**
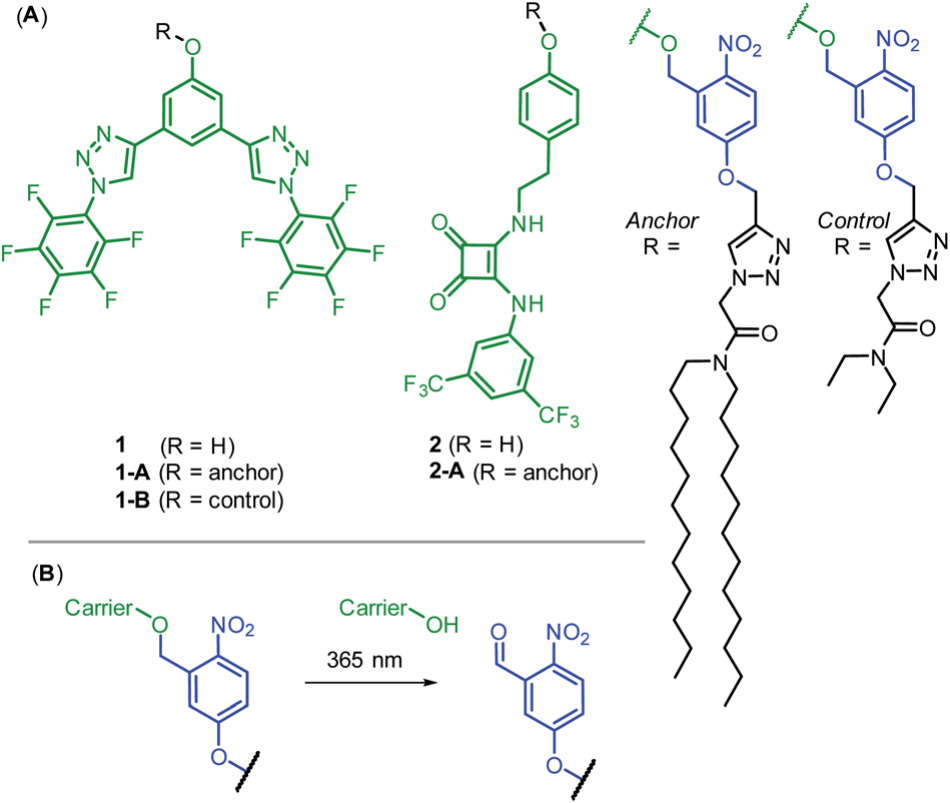
(A) Anion carriers 1 and 2, and their corresponding photo-responsive anchored derivatives 1-A and 2-A. 1-B acts as a control lacking the long dodecyl chains of the membrane anchor. (B) Photo-cleavage reaction of the *ortho*-nitrobenzyl linker.

We prepared two hydrogen bonding anion carriers 1 and 2, with pendant phenol functionality to enable facile attachment to the anchor whilst maintaining the desired neutrality of the carrier (Fig. 2A). Both carriers are based on known anion transport motifs: compound 1 employs two convergent CH hydrogen bonds for anion binding,^34^ whilst carrier 2 employs a squaramide motif as a bidentate NH hydrogen bond donor.^35^ Squaramide derivatives are well-established as highly potent anion carriers,^36,37^ including as pH-regulated transporters.^38^ However, to the best of our knowledge, methods to photo-cage their activity have not been reported. The corresponding anchored derivatives 1-A and 2-A were prepared by reaction of the pendant phenol with the 1-(bromomethyl)nitrophenol anchor precursor, to afford carriers connected to the anchor *via* a *ortho*-nitrobenzyl group,^39^ which is cleaved by irradiation with 365 nm light (Fig. 2B). This readily accessible and extensively studied photocleavable linker has previously been used to photo-cage the activity of protein ion channels^40–43^ and for single molecule mechanistic studies in nanopores.^44^ We also prepared control compound 1-B, in which the long dodecyl chains are replaced with the shorter ethyl groups, anticipated to be less effective at suppressing carrier mobility. Full synthetic schemes, procedures and characterisation for all compounds are available in the ESI.†

## Anion transport experiments

We first explored the chloride anion binding and transport properties of 1 and 2. As expected, ^1^H NMR anion binding titrations with tetrabutylammonium chloride revealed that both carriers bound chloride in *d*_6_-acetone, with a binding constants of log *K*_a_ = 3.0 for proto-triazole derivative 1, whilst the affinity to squaramide 2 was too high to be determined, even in an aqueous-acetone mixture (log *K*_a_ > 4, 98 : 2 *d*_6_-acetone : D_2_O).

The anion transport capabilities of 1 and 2 were determined in 1-palmitoyl-2-oleoyl-*sn*-glycero-3-phosphocholine large unilamellar vesicles (POPC LUVs, lipid concentration 31 μM), loaded with 8-hydroxypyrene-1,3,6-trisulfonate (HPTS, a pH-sensitive fluorophore) and buffered to pH 7.0 in NaCl solution. A pH gradient was applied across the membrane by addition of a base pulse (NaOH), followed by addition of the carrier as a DMSO solution (<0.5% v/v). The ability of the anionophore to dissipate the pH gradient by transmembrane Cl^−^/OH^−^ antiport (or the functionally equivalent H^+^/Cl^−^ symport) was determined by recording the change in the HPTS emission *I*_rel_ (*λ*_em_ = 510 nm) over time following excitation at *λ*_ex_ = 405/465 nm. At the end of each experiment, detergent (Triton X-100) was added to lyse the vesicles for calibration.

**Fig. 3:**
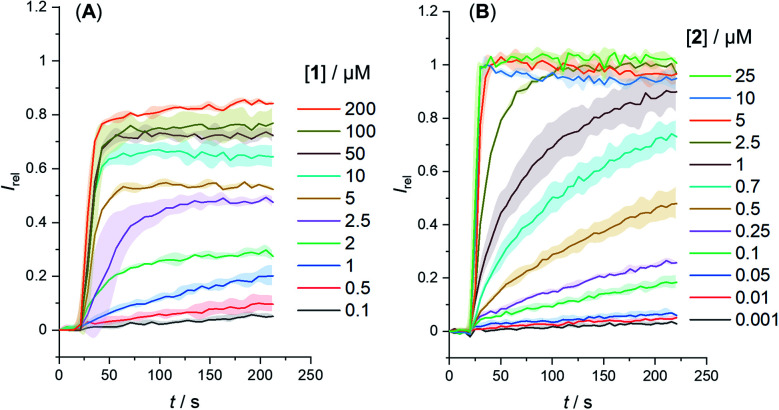
Change in ratiometric emission, *I*_rel_ (*λ*_em_ = 510 nm; *λ*_ex1_ = 405 nm, *λ*_ex2_ = 460 nm) upon addition of a NaOH base pulse (5 mM) to POPC LUVs (31 μM) containing 1 mM HPTS, 100 mM internal and external NaCl, buffered with 10 mM HEPES at pH 7.0. Data for (A) carrier 1 and (B) carrier 2. Errors denote standard deviations.

**Fig. 4:**
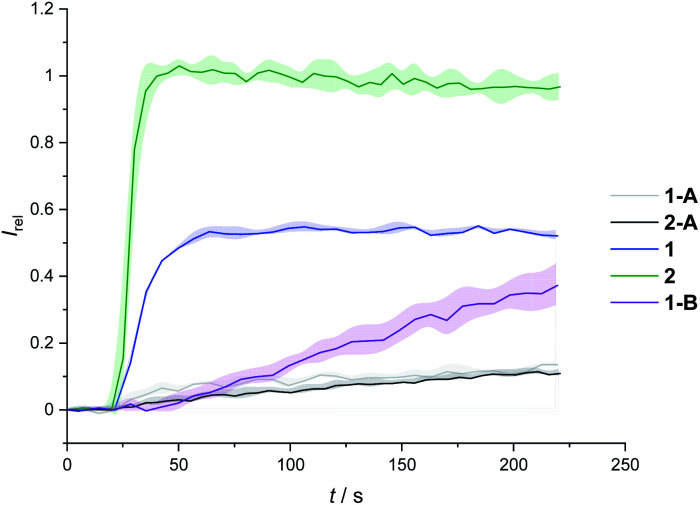
Transport activity of carriers 1 and 2, in comparison to their dodecyl-anchored analogues, 1-A and 2-A, and the ethyl-anchored control 1-B. Assay conditions as in Fig. 2, carrier concentration 5 μM.

We determined the concentration dependence of the activity of each carrier (Fig. 3A and B), and the corresponding dose response curves were fitted to the Hill equation to derive the effective concentration required to reach 50% activity in the given assay (EC_50_). Both carriers 1 and 2 proved to be effective anion transporters with EC_50_ values of 2.7 and 0.5 μM, respectively (Table 1).‡‡The transport assays plateau at *I*_rel_ < 1 for 1, compared to the more active carrier 2. This is likely due less efficient membrane uptake into all LUVs, as observed for related hydrophobic carriers.^34^Fig. 4 shows the transport data for 1 and 2 (5 μM), in comparison to their anchored analogues 1-A and 2-A, which are tethered *via* the *ortho*-nitrobenzyl linker to the dodecyl chains and are fully inactive. In contrast to the dodecyl anchored analogue 1-A, the corresponding short anchor derivative 1-B showed some activity. This demonstrates the key role of the long alkyl chains in 1-A and 2-A in suppressing the carrier mobility in the membrane, by maximizing van der Waals interactions with the lipids. Both anchored carriers 1-A and 2-A were too inactive to determine an EC_50_ value (EC_50_ > 100 μM).

**Table 1:** Anion transport data

Compound	EC_50_/μM^a^	*n* b
1	2.7 ± 0.6	1.1 ± 0.3
2	0.5 ± 0.03	2.3 ± 0.4
1-A	>100	—
2-A	>100	—
1 (*in situ* generation)	4.1 ± 0.3	3.9 ± 0.8
2 (*in situ* generation)	2.7 ± 0.2	1.5 ± 0.1

a Effective concentration to reach 50% of maximal activity in the HPTS assay, in LUVs of POPC (mean diameter 200 nm, lipid concentration 31 mM) loaded with 1 mM HPTS, NaCl (100 mM) and buffered at pH 7.0 with 10 mM HEPES. For compounds of low activity, estimated lower bounds for the EC_50_ value are given.

b Hill coefficient.

Inactivity of the system when the chloride anions in the buffer were exchanged for gluconate – a large hydrophilic anion too hydrophilic to be transported – confirmed that the transport in the presence of chloride is cation independent, occurring *via* Cl^−^/OH^−^ antiport (or Cl^−^/H^+^ symport) and not *via* a cation-dependent H^+^/Na^+^ antiport mechanism (Fig. S50†). Inactivity of 1 and 2 and their anchored analogues 1-A and 2-A in this assay, as well as in the carboxyfluorescein (CF) leakage assay (Fig. S52†), rules out potential membrane disruption and non-specific ion leakage as being responsible the dissipation of the transmembrane pH gradient. Inactivity of 1 in the gel phase of dipalmitoylphosphatidylcholine (DPPC) lipids at 25 °C, and restoration of activity at 45 °C, above the gel–liquid phase transition temperature (*T*_m_ = 41 °C), confirms a carrier mechanism rather than self-assembly of the hydrophobic planar 1 into membrane spanning channels, in agreement with results for similar carriers (Fig. S53†).^34^

## Photo-activation of ion transport

**Fig. 5:**
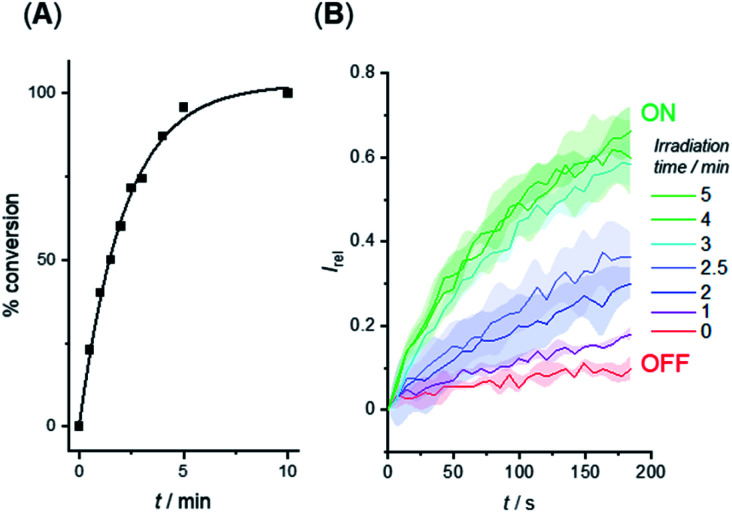
(A) Generation of carrier 1 from 1-A in DMSO solution by irradiation with 365 nm LED (1.3 W). (B) Switch-ON transport triggered by *in situ* photo-generation of carrier 1 from 1-A in the membrane of LUVs. Separate samples of LUVs containing 1-A were irradiated for the indicated duration prior to the addition of the base pulse to start the transport experiment. Assay conditions as in Fig. 3, anchored carrier concentration 5 μM.

We investigated the photo-triggered cleavage of the anchor from 1-A and 2-A initially *via*^1^H NMR experiments. A 2 mM sample of each anchored carrier in DMSO was subjected to irradiation at 365 nm using an LED (irradiation intensity 1.3 W), and the conversion to the free carrier was determined after increasing irradiation time intervals. Under these conditions, rapid cleavage was observed for both carriers: the half-life for the photo–cleavage reaction and generation of free carrier 1 from 1-A was 1.5 minutes (Fig. 5A), and comparable photo-generation of 2 from 2-A was also achieved under the same conditions (Table S1†).

Given the rapid photo-generation of free carrier in solution, we explored the possibility of generating the carrier in LUVs *in situ*; that is from the anchored carrier already incorporated into the membrane of vesicles. In an initial experiment, we added 1-A (5 μM) in DMSO to LUVs containing HPTS and then subjected the sample to the pH dissipation assay conditions by addition of the base pulse (Fig. 5B, red data). At this concentration and prior to irradiation the anchored carrier is inactive, and no anion transport was observed. Separate samples of vesicles containing 1-A (5 μM) were then irradiated using the 365 nm LED for increasing periods of time, and the transport activity determined (Fig. 5B). Maximum activity, comparable to that obtained by direct addition of 1, was reached after ∼3 minutes, indicating that complete de-caging of 1-A in the membrane is achieved over a timescale consistent with results from solution phase de-caging experiments.

**Fig. 6:**
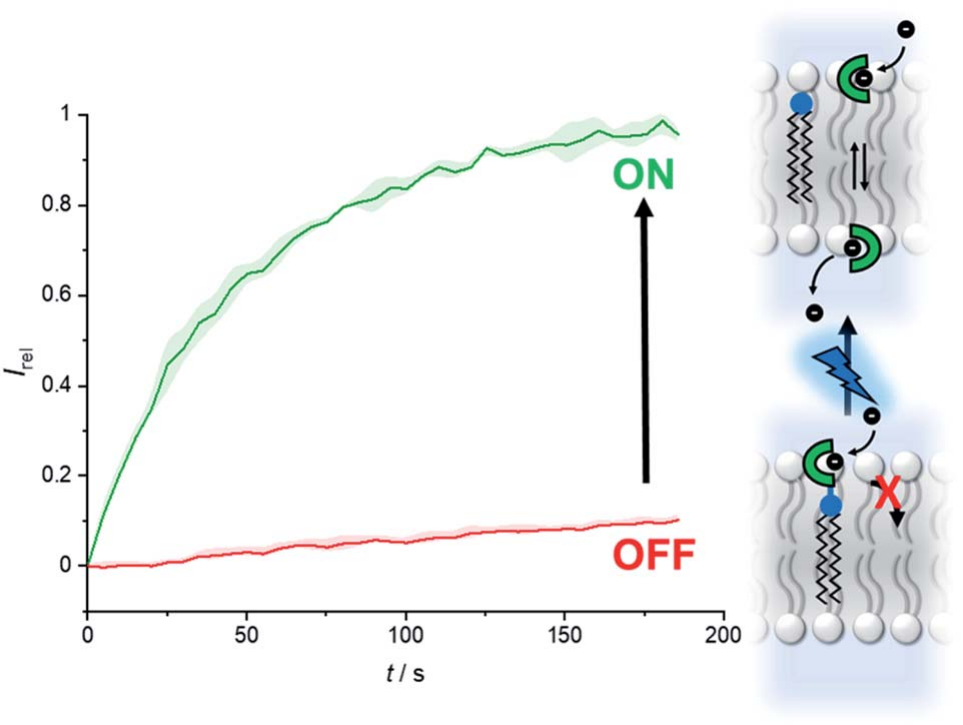
Generation of carrier 2 from 2-A by irradiation with 365 nm LED (1.3 W) to switch on anion transport. Assay conditions as in Fig. 2, anchored carrier concentration 5 μM.

Fig. 6 shows the analogous switch-ON experiment for 5 μM 2-A in LUVs, following 3 minutes of *in situ* irradiation with 365 nm. Again, highly effective switch-ON transport is observed, from an OFF state with negligible background activity. The enhancement in activity corresponds to a 30-fold increase in transport kinetics under these conditions. Inactivity in the carboxyfluorescein leakage assay or HPTS assay with gluconate following irradiation rules out photo-induced membrane disruption leading to the observed ion transport. These results for both anchored carriers demonstrate that a membrane anchor connected *via* a photo-cleavable linker is an effective strategy for enabling effective OFF–ON activation of transport activity.

The concentration dependence of the transport data following *in situ* irradiation for 3 minutes was determined for both 1-A and 2-A, affording EC_50_ values of 4.1 μM and 2.7 μM, respectively. These are comparable to those obtained by direct addition of the free carriers 1 and 2 (EC_50_ = 2.7 μM and 0.5 μM, respectively, Table 1). The small variation in EC_50_ values between the *in situ* generated and externally added free carriers, and the subtle differences in concentration dependence of transport rates (characterised by the Hill coefficient, *n*) presumably arise from differences in membrane delivery of the anchored carriers compared to the free carrier analogues, given that photo-irradiation under these conditions quantitatively releases the carriers from their anchor within the membrane.

In both the anchored and free carrier states, the anion binding site is conserved. Differences in binding affinity between anchored (OFF state) and free carriers (ON state) can therefore be ruled out as being responsible for the observed dramatic difference in activities between the two. Variation in delivery to the membrane can also be ruled out as factor, because the *in situ* generation of 1 and 2 occurs from anchored analogues that are already present in the membrane. The observed OFF–ON switching of activity must therefore arise from the suppressed mobility of the carrier in the anchored state, and restoration of its mobility of the photo-generated free carrier. Importantly, this mechanism of photo-caging transporters does not require modification of the ion binding site; needing only attachment of the photo-caged anchor to a remote position on the carrier. We therefore anticipate that this method of photo-regulating activity will be applicable to a broad range of transmembrane molecular transporters.

## Conclusions

We report a new mechanism for OFF–ON switching of activity of supramolecular mobile anion carriers that exploits photo-regulated carrier mobility. Anion carrier mobility through the membrane is prerequisite for activity, because the carrier must approach both sides of the membrane to bind, transport and release the ion. By appending long alkyl chains to a mobile carrier *via* a photo-cleavable linker, carrier mobility is suppressed, switching-OFF transport. Photo-irradiation of the anchored carrier in the membrane releases the free carrier, restoring its mobility and switching on anion transport. The ability to regulate synthetic ion transporters with external stimuli is crucial for spatio-temporal targeting of transport activity, for example for targeted therapeutics or tools for chemical biology. The method reported here allows for effective switching between an OFF state with negligible background activity to an active ON state, using a simple *ortho*-nitrobenzyl cleavable linker and does not involve modification of the ion binding site; requiring only attachment of the photo-caged anchor to a remote position on the carrier. We therefore anticipate that this method of photo-regulating activity will be general for a broad range of ion transporters, as well as a range of different stimuli through the use of alternative cleavable linker designs.

## Author contributions

L. E. B. carried out the experimental work. M. J. L. conceived and supervised the project. Both authors analysed the data and wrote the article.

## Conflicts of interest

There are no conflicts to declare.

## Supplementary Material

SC-013-D2SC03322D-s001
