# Supplementary material for: Controlling transmembrane ion transport via photo-regulated carrier mobility
Source: Chem Sci. 2022 Jul 8;13(33):9531–6. doi: 10.1039/d2sc03322d (PMC9400602; doi:10.1039/d2sc03322d)
Supplement: SC-013-D2SC03322D-s001 [file SC-013-D2SC03322D-s001.pdf]

Electronic Supplementary Material (ESI) for Chemical Science.
This journal is © The Royal Society of Chemistry 2022

Supporting information for

Controlling transmembrane ion transport via photo-regulated carrier mobility

Laura E. Bickerton and Matthew J. Langton*[a]

[a]Chemistry Research Laboratory, University of Oxford, Mansfield Road, Oxford, OX1 3TA, UK

Contents

1. **Materials and Methods** 2

2. **Synthesis and Characterization** 2

3. **^{1}H NMR Titration Experiments** 21

4. **Photocleavage Experiments** 22

5. **Anion Transport Experiments** 25

   Vesicle Preparation 25

   The HPTS Assay[11,12] 25

   Sodium Gluconate HPTS Assay 29

   Carboxyfluorescein Leakage Assay[13] 30

   HPTS Assay with DPPC lipids 30

6. **References** 31

1. Materials and Methods

All reagents and solvents were purchased from commercial sources and used without further purification. Lipids were purchased from Avanti polar lipids and used without further purification. Where necessary, solvents were dried by passing through an MBraun MPSP-800 column and degassed with nitrogen. Column chromatography was carried out on Merck® silica gel 60 under a positive pressure of nitrogen. Where mixtures of solvents were used, ratios are reported by volume. TEA was distilled and stored over potassium hydroxide. NMR spectra were recorded on a Bruker AVIII 400, Bruker AVII 500 (with He cryoprobe) and Bruker AVIIIHD 500 spectrometers. Chemical shifts are reported as δ values in ppm. Mass spectra were carried out on an Agilent 6120 bench-top single quadrupole, a Waters LCT Premier XE benchtop (oa-TOF) and a Thermo Exactive High-Resolution Orbitrap FTMS spectrometer. Fluorescence spectroscopic data were recorded using a Horiba Duetta fluorescence spectrophotometer, equipped with Peltier temperature controller and stirrer. Experiments were conducted at 25 °C unless otherwise stated. Vesicles were prepared as described below using Avestin "LiposoFast" extruder apparatus, equipped with polycarbonate membranes with 200 nm pores. GPC purification of vesicles was carried out using GE Healthcare PD-10 desalting columns prepacked with Sephadex G-25 medium.

2. Synthesis and Characterization

General comments

Tris[(1-benzyl-1*H*-1,2,3-triazol-4-yl)methyl]amine (TBTA),[1] 1,3-diethynylphenol **3**,[2] azido-pentafluorobenzene **4**,[3] 2-nitro-5-(prop-2-yn-1-yloxy)benzaldehyde **5**,[4] (2-nitro-5-(prop-2-yn-1-yloxy)phenyl)methanol **6**,[5] 2-azido-*N*,*N*-didodecylacetamide **9**,[6] and 2-azido-*N*,*N*-diethylacetamide **10**,[7] were all prepared according to literature procedures.

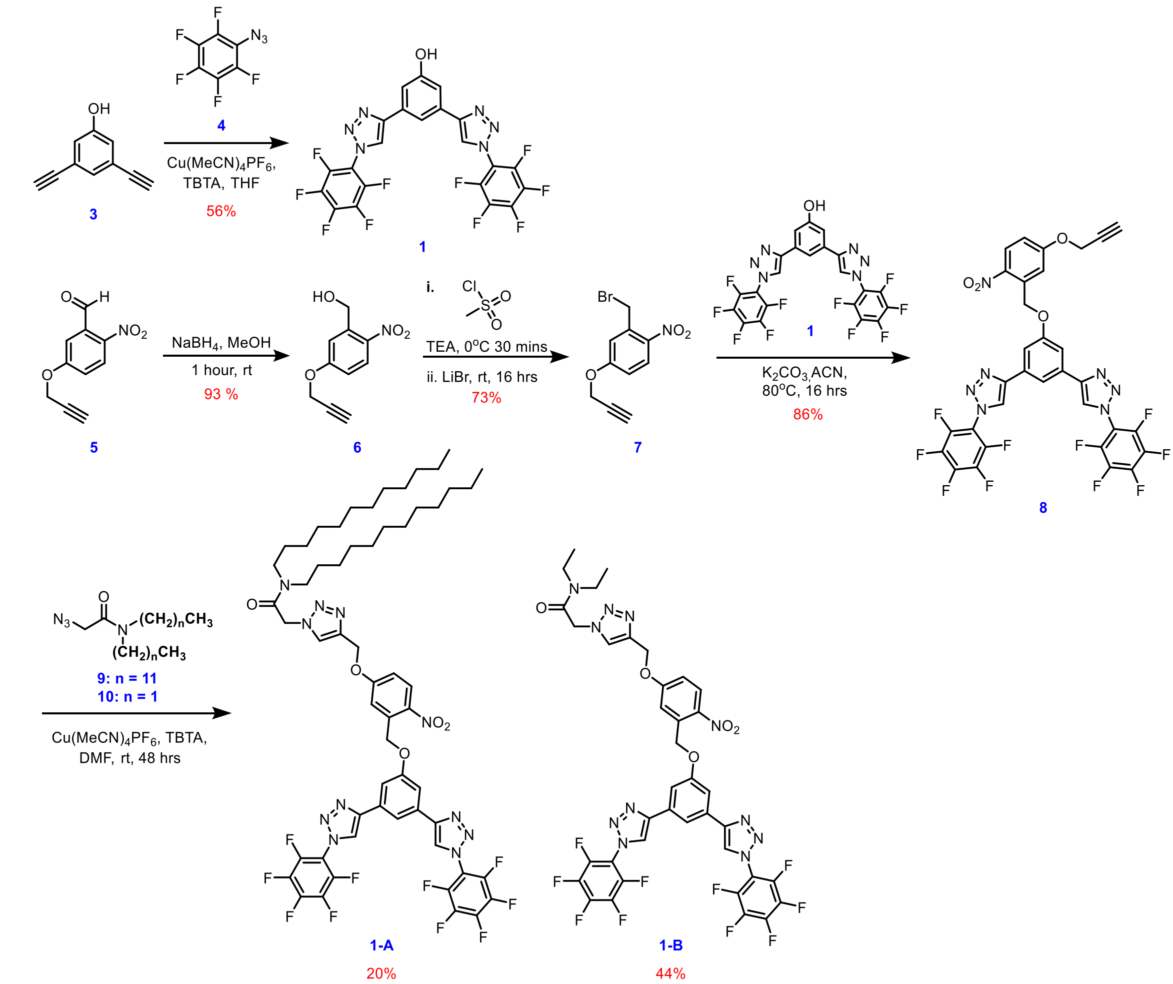

Scheme S1. Synthesis of **1, 1-A** and **1-B**.

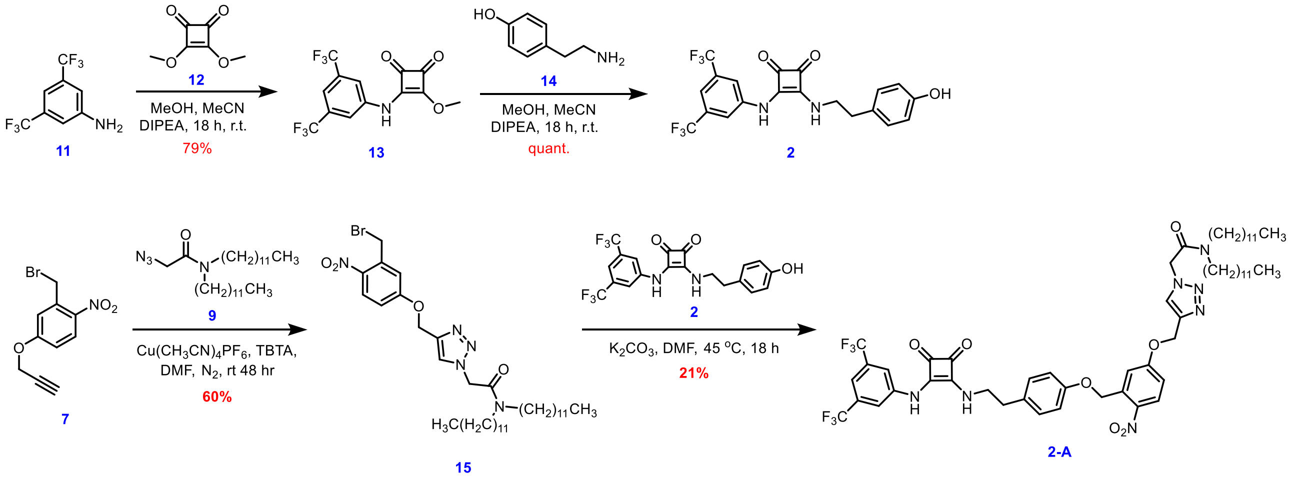

Scheme S2. Synthesis of **2** and **2-A.**

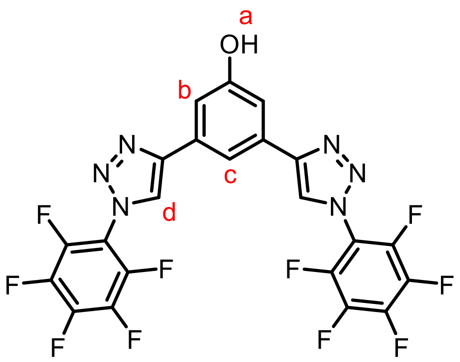

Carrier 1. $Cu(CH_3CN)_4PF_6$ (105 mg, 0.282 mmol, 0.5 eq.) and TBTA (75.0 mg, 0.141 mmol, 0.25 eq.) were stirred in dry degassed THF (5 mL), followed by the addition of 3,5-diethynylphenol **3** (80.0 mg, 0.563 mmol, 1 eq.) with azido-pentafluorobenzene **4** (706 mg, 3.38 mmol, 6 eq.). The reaction was stirred under N_2 for 48 hours. The solvent was removed in vacuo, and the residue dissolved in chloroform (20 mL). The organic mixture was washed with 10% aqueous EDTA/NH_4OH solution (2 x 10 mL), followed by brine (20 mL) and then dried over $MgSO_4$. The solvent was removed in vacuo to give the crude product. The product was purified by column chromatography (30% EtOAc in hexane) to give a white solid (197 mg, 62%). ^{1}H NMR (600 MHz, DMSO) δ 10.02 (s, 1H$_a$), 9.26 (s, 2H$_d$), 7.99 (q, J = 1.8 Hz, 1H$_c$), 7.42 (d, J = 1.5 Hz, 2H$_b$). ^{13}C NMR (126 MHz, DMSO) δ 158.64, 146.69, 141.84, 141.70, 137.60, 131.23, 124.65, 113.66, 112.41.^{19}F NMR (565 MHz, DMSO) δ -147.24 (s, 4F), -151.22 (t, J = 23.0 Hz, 2F), -160.82 (quintet, J = 21.2 Hz, 4F). HRMS-ESI (m/z) Calculated for $C_{22}H_7ON_6F_{10}$-H^+: 561.0516; found: 561.0514.

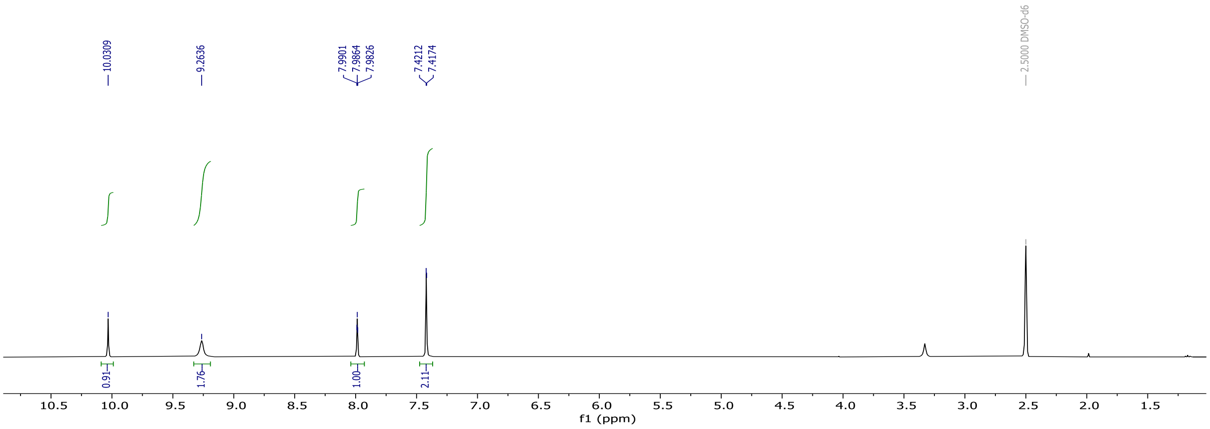

Figure S1. ^{1}H NMR Spectrum of **1** (DMSO-d_6, 298 K, 600 MHz).

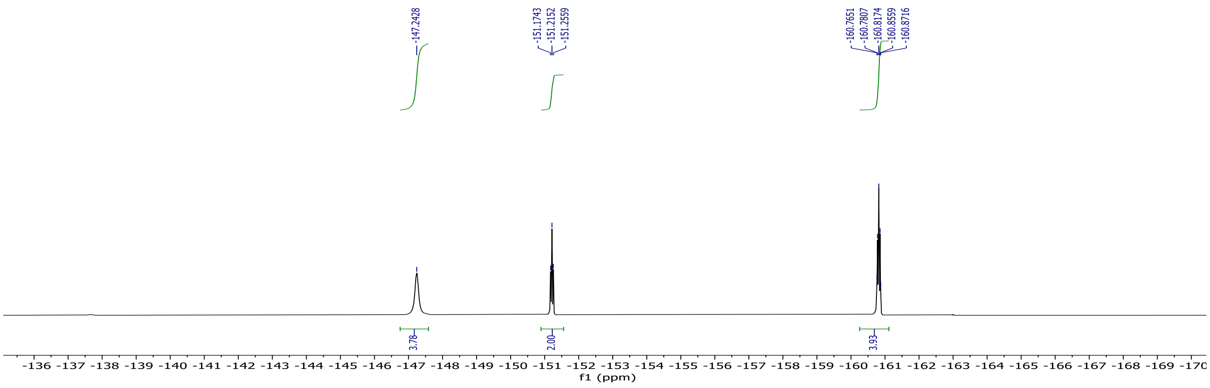

Figure S2. ^{19}F NMR Spectrum of **1** (DMSO-d_6, 298 K, 565 MHz).

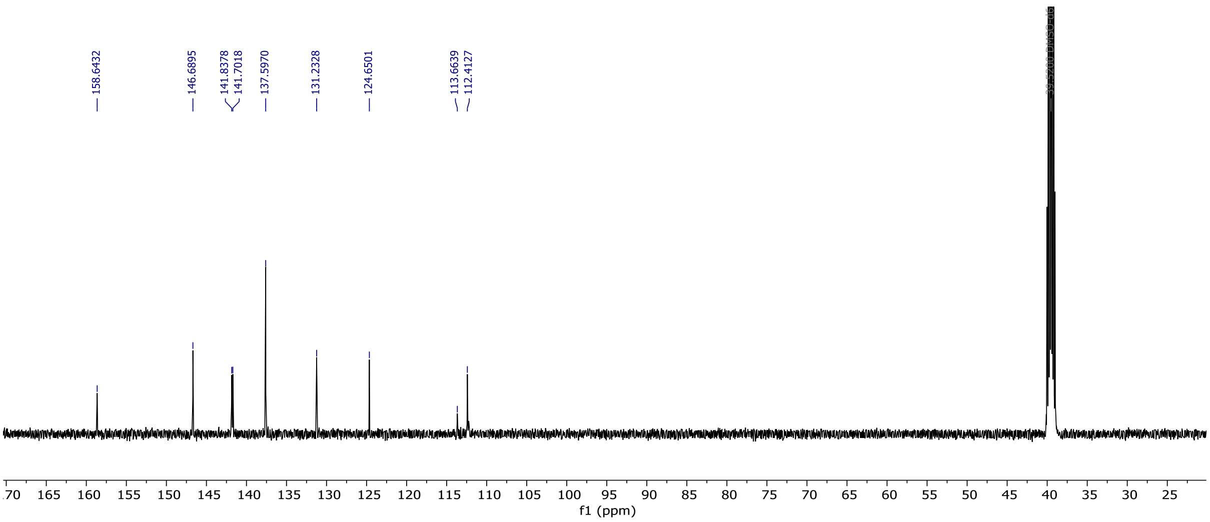

Figure S3. ^{13}C NMR Spectrum of **1** (DMSO-d_6, 298 K, 126 MHz).

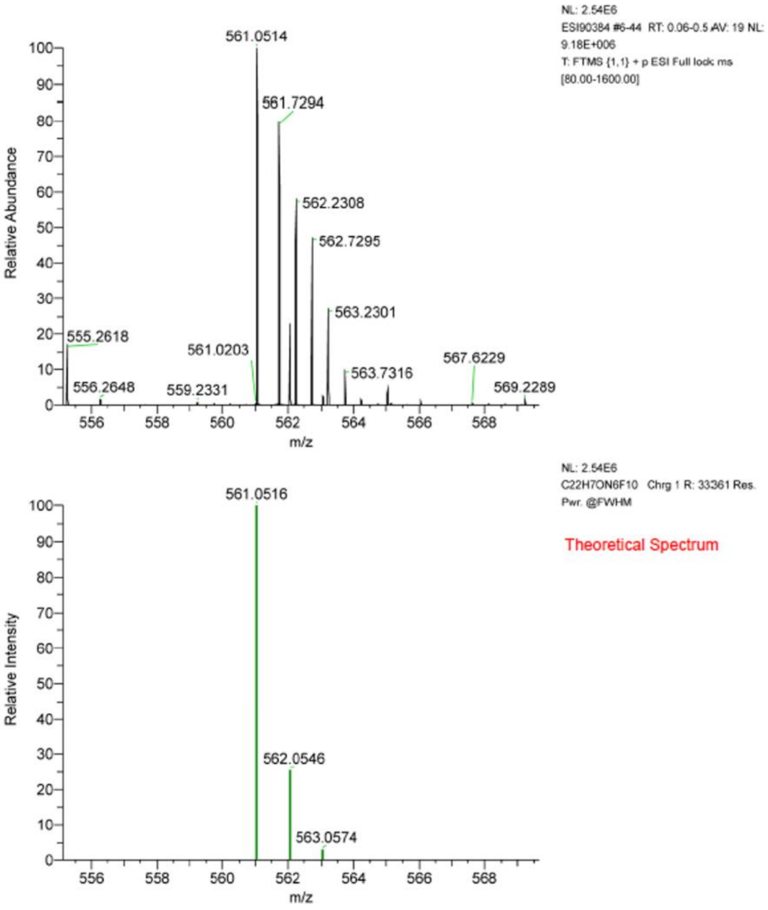

Figure S4. HRMS-ESI spectrum of compound **1**.

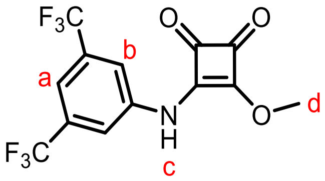

Compound 13. Synthesised according to literature procedures as follows;[8] 3,5-bis(trifluoromethyl)aniline **11** (1.015 g, 4.437 mmol, 0.9 eq.) and 3,4-dimethoxy-3-cyclo-butene-1,2-dione **12** (700 mg, 4.9296 mmol 1 eq.) were stirred in MeOH (20 mL) at rt overnight. The solid residue was washed with cold MeOH (20 mL) then dissolved in MeCN (minimum) and adsorbed onto silica. The dry loaded compound was eluted with DCM to afford the product as a pure white solid. (1.23 g, 82%). ^{1}H NMR (600 MHz, DMSO) δ 11.17 (s, 1H$_c$), 8.03 (s, 2H$_b$), 7.75 (s, 1H$_a$), 4.41 (s, 3H$_d$). ^{19}F NMR (565 MHz, DMSO) δ -61.82 (s, 6F). ^{13}C NMR (126 MHz, DMSO) δ 187.40, 184.49, 179.92, 169.14, 140.22, 131.18, 123.08, 119.31, 116.27, 60.95. HRMS-ESI (m/z) Calculated for $C_{13}H_6O_3NF_6$-H^+: 338.0246; found 338.0251.

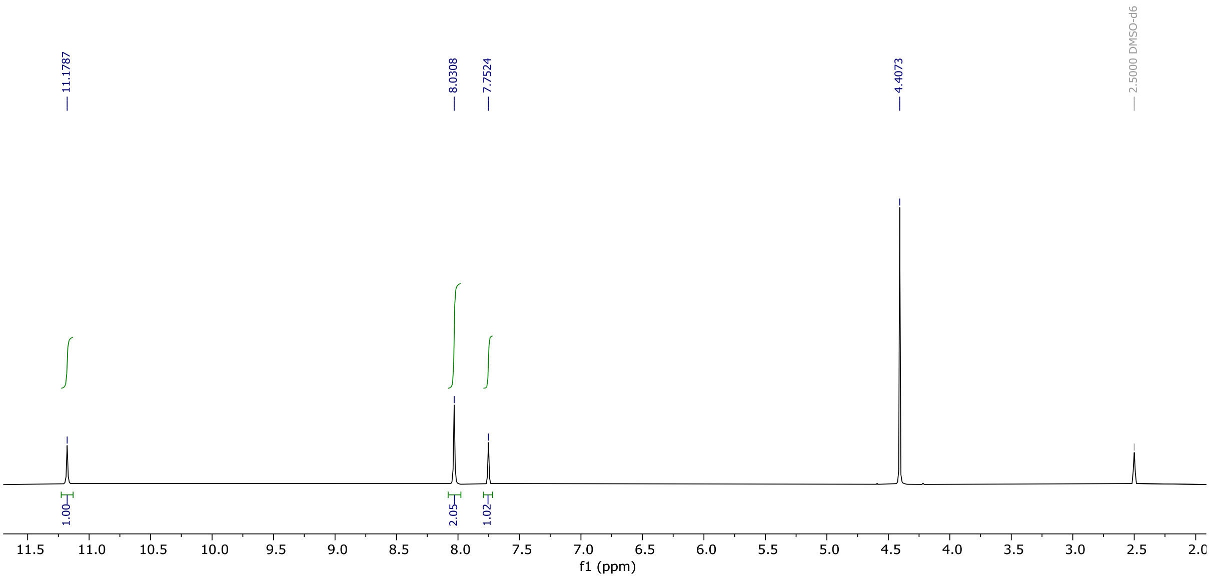

Figure S5. ^{1}H NMR Spectrum of **13** (DMSO-d_6, 298 K, 600 MHz).

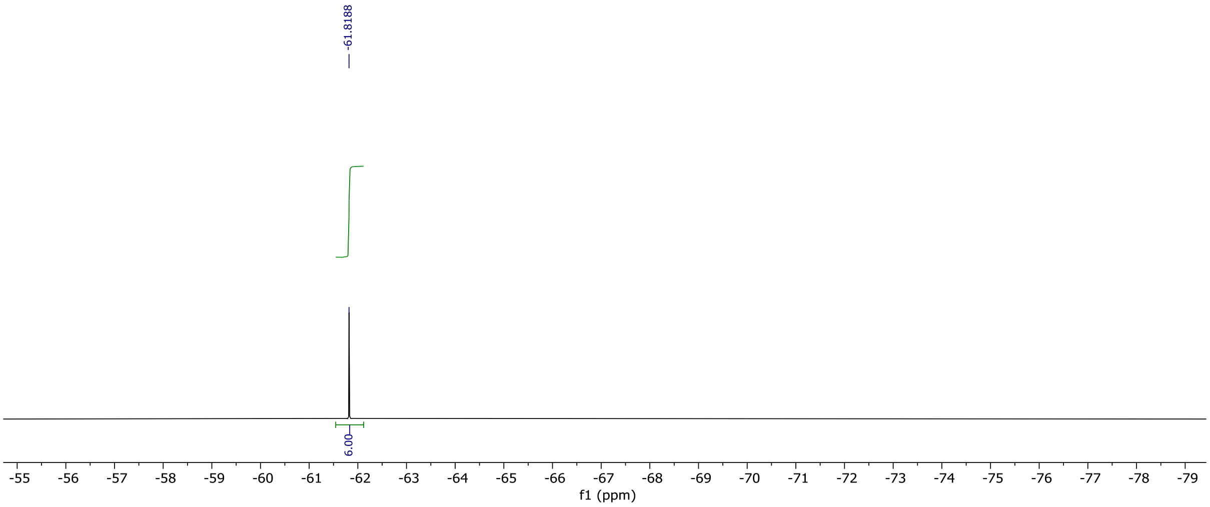

Figure S6. ^{19}F NMR Spectrum of **13** (DMSO-d_6, 298 K, 565 MHz).

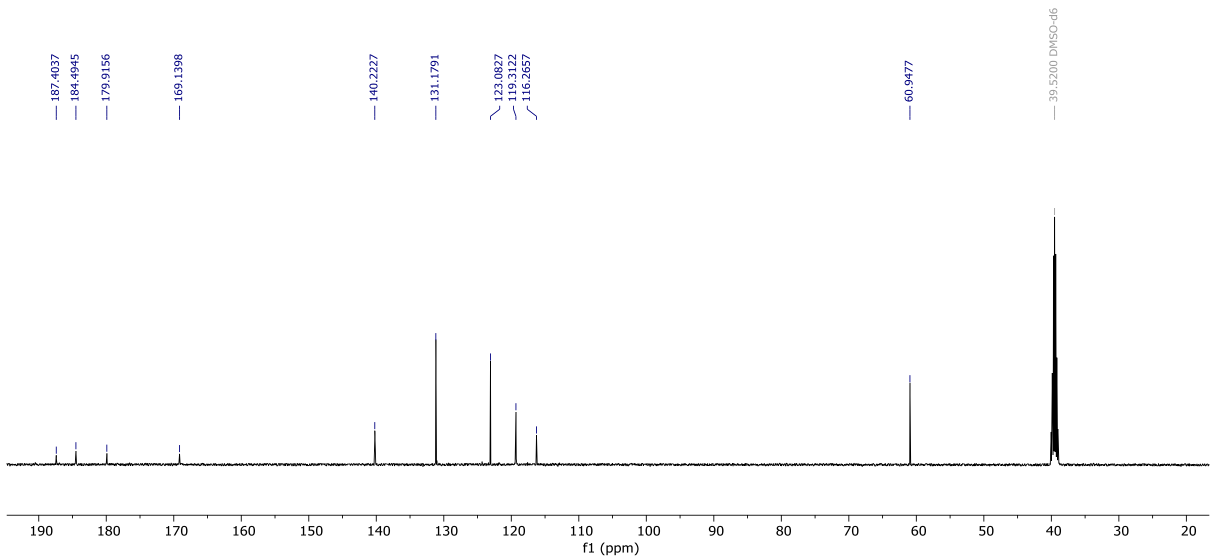

Figure S7. ^{13}C NMR Spectrum of **13** (DMSO-d_6, 298 K, 126 MHz).

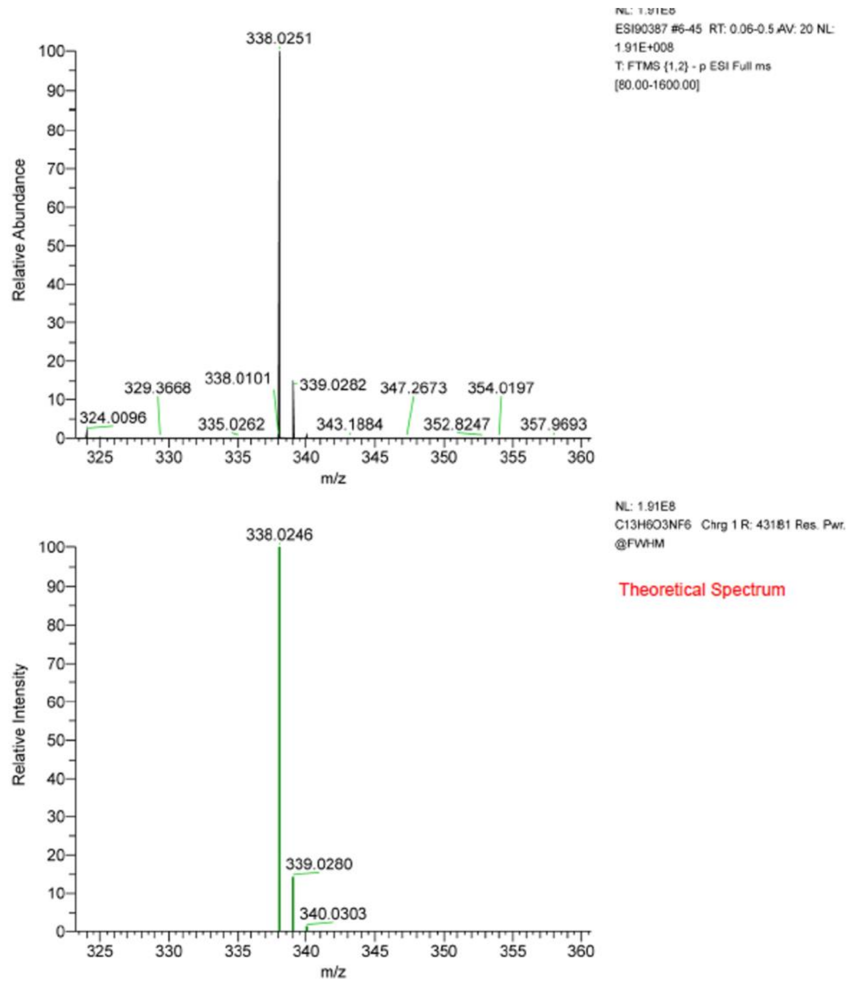

Figure S8. HRMS-ESI spectrum of compound **13**.

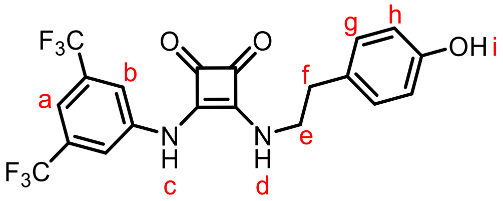

Carrier 2. Tyramine **14** (100 mg, 0.295 mmol, 1 eq.) was dissolved in MeOH (5 mL) and treated with dropwise additions of DIPEA (114 mg, 0.885 mmol, 3 eq.). A solution of compound **13** (40 mg, 0.295 mmol, 1 eq.) in MeCN (5 mL) was added and the reaction was stirred at rt overnight. The solid residue was washed with cold MeOH (5 mL) and MeCN (5 mL) to afford the pure product (109 mg, 83%). ^{1}H NMR (600 MHz, DMSO) δ 10.14 (s, $1H_c$), 9.21 (s, $1H_i$), 8.01 (s, $2H_b$), 7.68 (s, $1H_d$), 7.62 (s, $1H_a$), 7.05 (d, *J* = 7.9 Hz, $2H_g$), 6.70 (d, *J* = 7.9 Hz, $2H_h$), 3.81 (d, *J* = 7.0 Hz, $2H_e$), 2.78 (t, *J* = 7.1 Hz, $2H_f$). ^{19}F NMR (565 MHz, DMSO) δ -61.81 (s, 6F). ^{13}C NMR (126 MHz, DMSO) δ 184.72, 180.35, 169.75, 162.28, 155.96, 141.12, 131.36, 129.73, 128.16, 123.18, 117.95, 115.28, 114.64, 45.44, 35.99. HRMS-ESI (m/z) Calculated for $C_{20}H_{15}O_3N_2F_6$-H^+: 445.0981; found 445.0982.

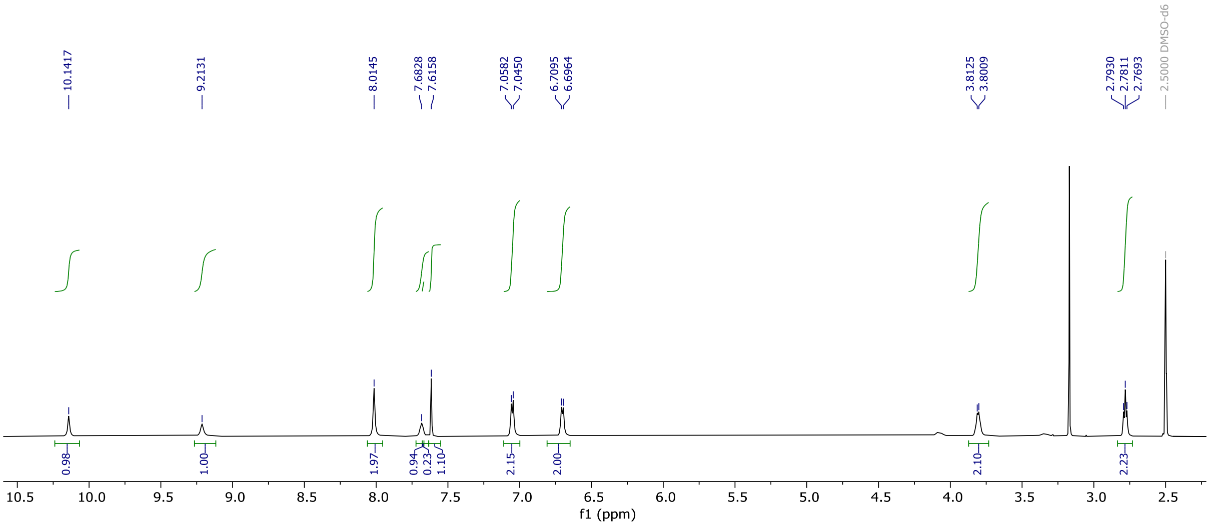

Figure S9. ^{1}H NMR Spectrum of **2** (DMSO-d_6, 298 K, 600 MHz).

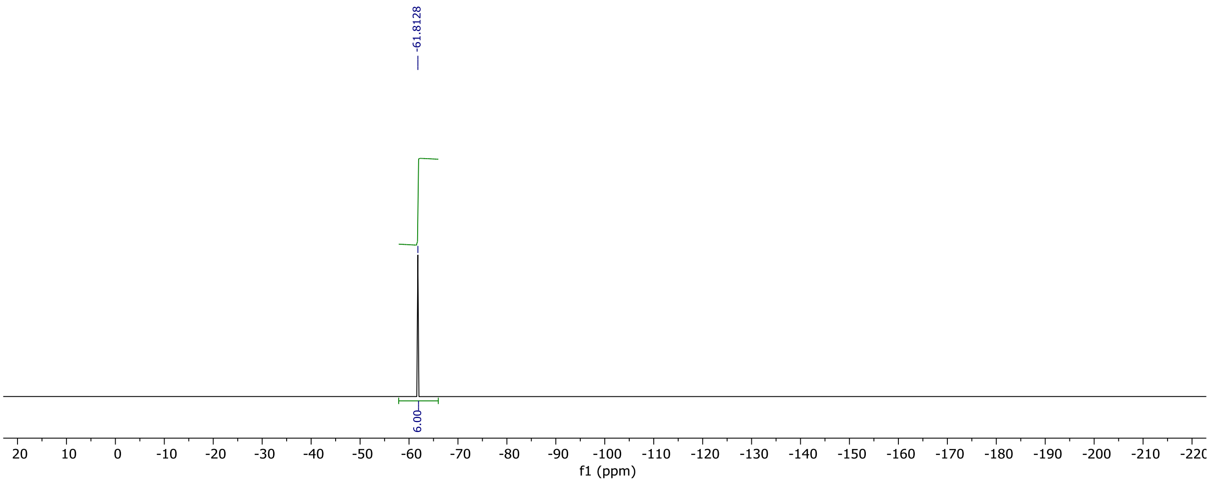

Figure S10. ^{19}F NMR Spectrum of **2** (DMSO-d_6, 298 K, 565 MHz).

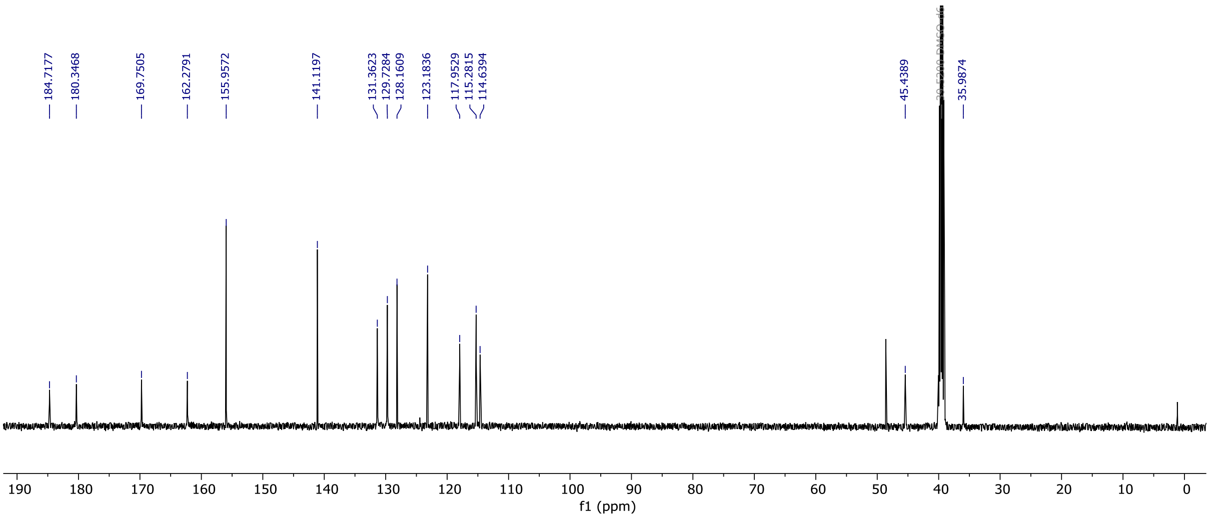

Figure S11. ^{13}C NMR Spectrum of **2** (DMSO-d_6, 298 K, 126 MHz).

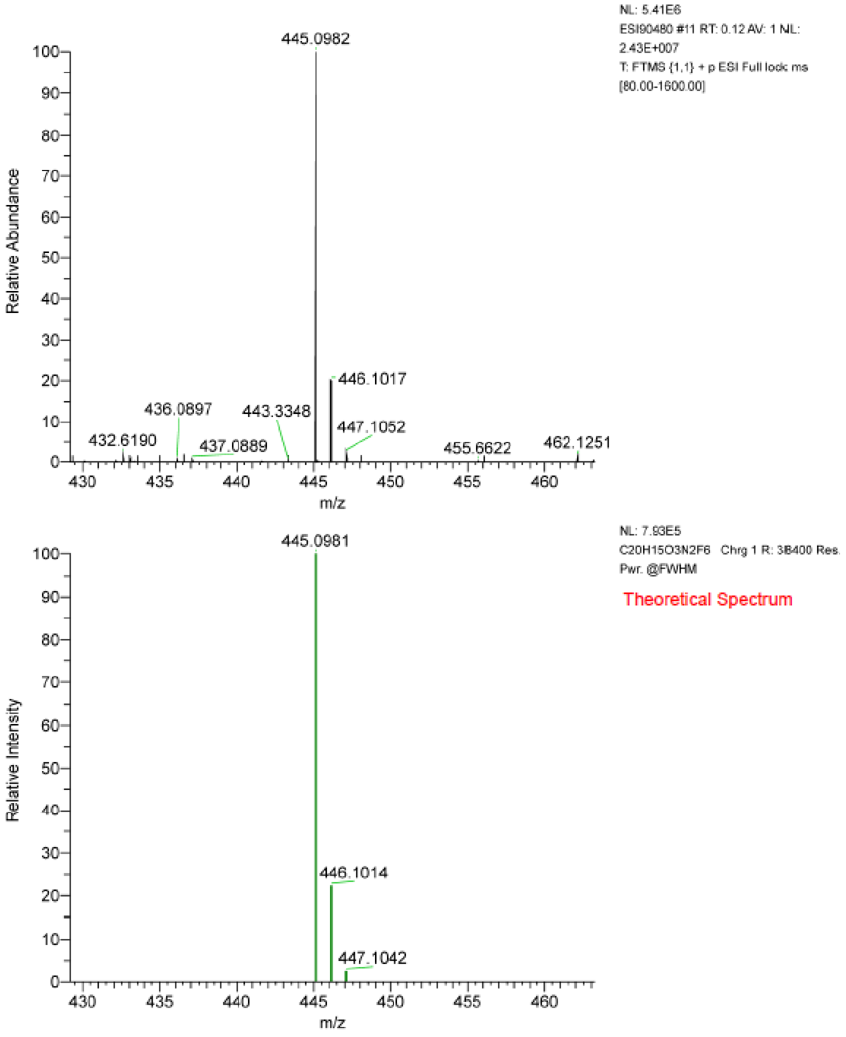

Figure S12. HRMS-ESI spectrum of compound **2**.

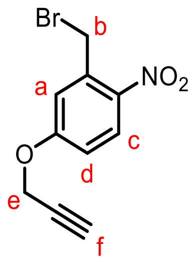

Compound 7. (2-nitro-5-(prop-2-yn-1-yloxy)phenyl)methanol **6** (3.35 g, 0.0162 mol, 1 eq.) was dissolved in DCM (50 mL), cooled to 0 °C, and treated with TEA (4.5 mL, 0.032 mol, 2 eq.) and methanesulfonyl chloride (2.78 g, 0.0243 mol, 1.5 eq.). The reaction mixture was stirred for 30 mins then washed with sat. Na_2CO_3 solution (2 x 20 mL) and brine (2 x 20 mL) then dried with $MgSO_4$ and the solvent was removed *in vacuo*. The resulting solid was stirred in THF (50 mL) with LiBr (5.62 g, 0.0647 mol, 4 eq.) overnight at rt. The reaction mixture was concentrated *in vacuo* then diluted with DCM and washed with water (2 x 20 mL) and brine (2 x 20 mL) followed by drying over $MgSO_4$. The crude product was purified by silica gel flash chromatography using DCM as the eluent to afford the product as a yellow solid (3.23 g, 74%). ^{1}H NMR (600 MHz, $CDCl_3$) δ 8.14 (d, *J* = 9.1 Hz, 1H$_c$), 7.12 (d, *J* = 2.8 Hz, 1H$_a$), 7.02 (dd, *J* = 9.1, 2.8 Hz, 1H$_d$), 4.85 (s, 2H$_b$), 4.80 (d, *J* = 2.4 Hz, 2H$_e$), 2.60 (t, *J* = 2.4 Hz, 1H$_f$). ^{13}C NMR (151 MHz, $CDCl_3$) δ 161.26, 141.48, 135.74, 128.37, 118.77, 114.98, 56.50, 29.75.

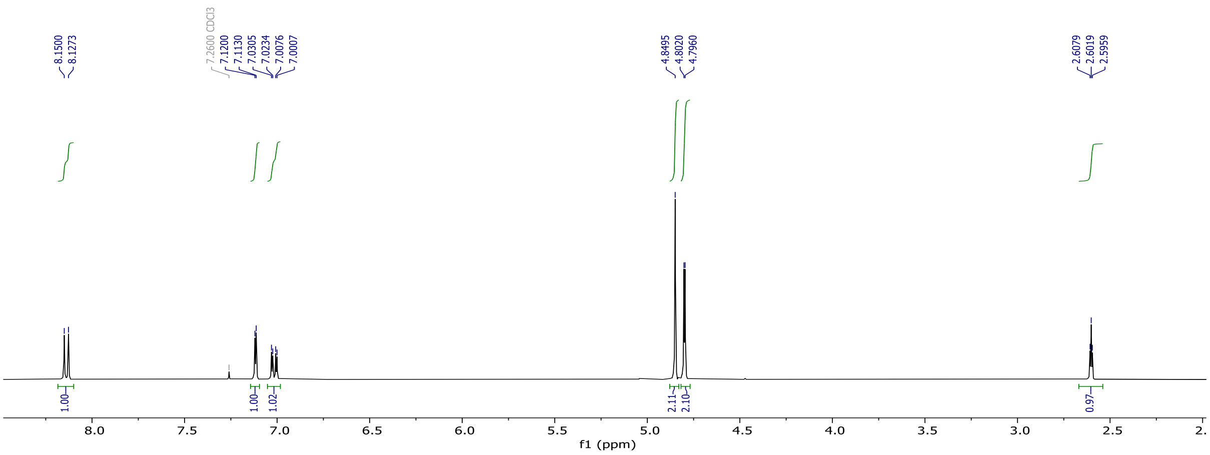

Figure S13. ^{1}H NMR Spectrum of **7** ($CDCl_3$, 298 K, 600 MHz).

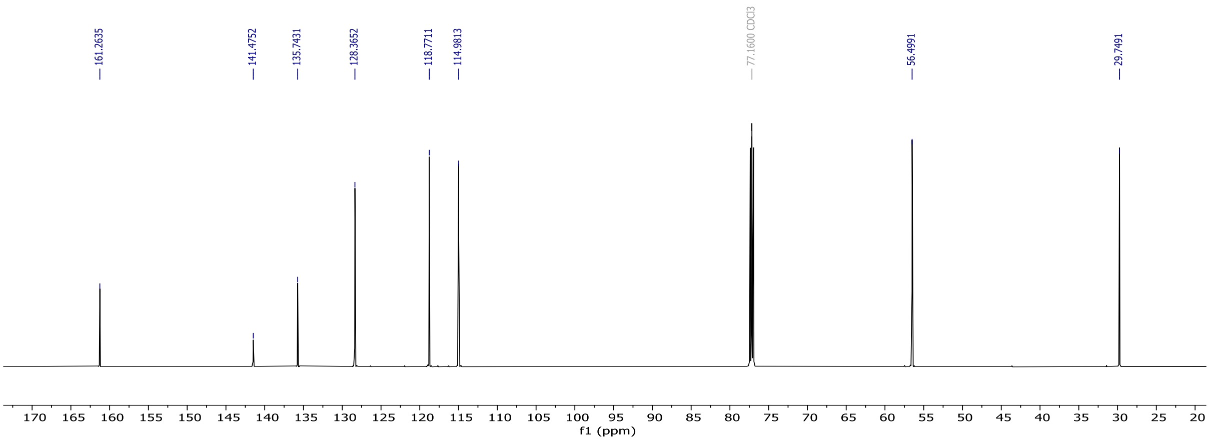

Figure S14. ^{13}C NMR Spectrum of **7** ($CDCl_3$, 298 K, 151 MHz).

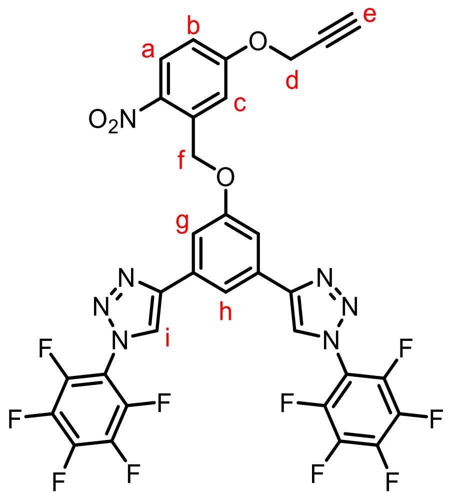

Compound 8. Carrier **1** (56 mg, 0.10 mmol, 1 eq.) and K_2CO_3 (30 mg, 0.22 mmol, 2.2 eq.) were stirred in MeCN (7.5 mL) at 0 °C. The reaction mixture was warmed to rt, compound **7** (54 mg, 0.20 mmol, 2 eq.) was added then the reaction was stirred overnight at 45 °C. The reaction mixture was concentrated in vacuo then diluted with DCM (15 mL) and washed with water (1 x 10 mL) and brine (1 x 10 mL) followed by drying over $MgSO_4$. The crude product was purified by silica gel flash chromatography using DCM as the eluent to afford the solid product (38 mg, 51%). ^{1}H NMR (500 MHz, $CDCl_3$) δ 8.28 (d, *J* = 9.1 Hz, 1H_a), 8.18 (s, 2H_i), 8.08 (t, *J* = 1.4 Hz, 1H_h), 7.62 (d, *J* = 1.5 Hz, 2H_g), 7.55 (d, *J* = 2.8 Hz, 1H_c), 7.04 (dd, *J* = 9.1, 2.9 Hz, 1H_b), 5.68 (s, 2H_f), 4.82 (d, *J* = 2.4 Hz, 2H_d), 2.56 (t, *J* = 2.4 Hz, 1H_e). ^{19}F NMR (471 MHz, $CDCl_3$) δ -144.81 – -146.35 (m, 4F), -149.49 (t, *J* = 21.7 Hz, 2F), -158.53 – -159.63 (m, 4F). ^{13}C NMR (126 MHz, $CDCl_3$) δ 162.16, 159.38, 147.61, 142.58, 140.52, 138.30, 136.79, 131.82, 128.09, 122.63, 117.06, 114.43, 114.13, 113.06, 113.02, 67.48, 56.53, 29.85. HRMS-ESI (m/z) Calculated for $C_{32}H_{14}O_4N_7F_{10}$-H^+: 750.0942; found 750.0934.

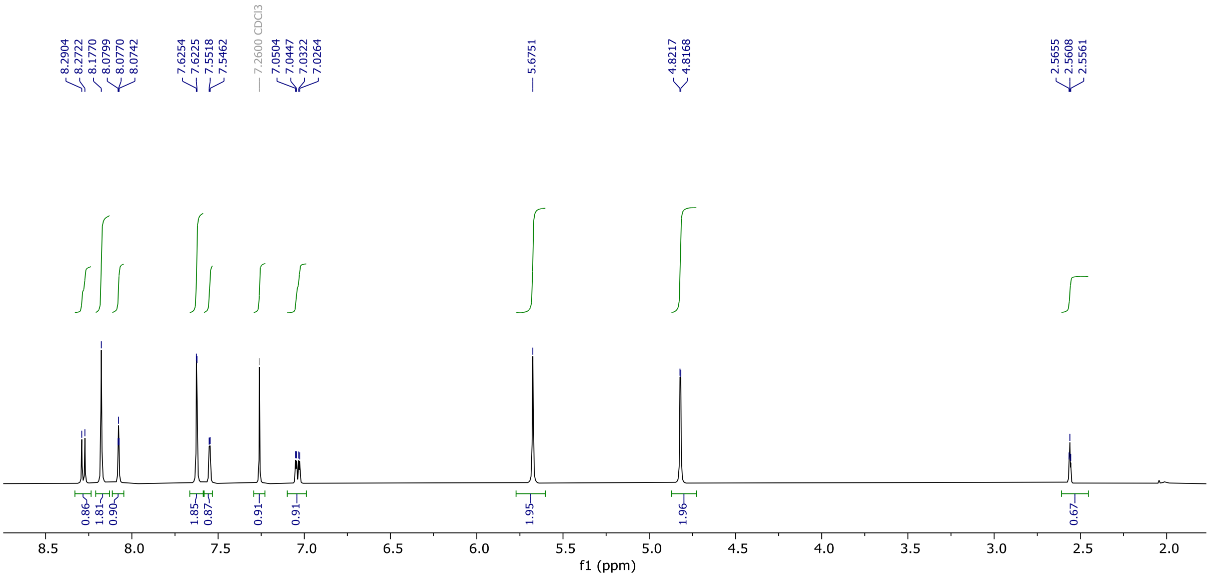

Figure S15. ^{1}H NMR Spectrum of **8** ($CDCl_3$, 298 K, 500 MHz).

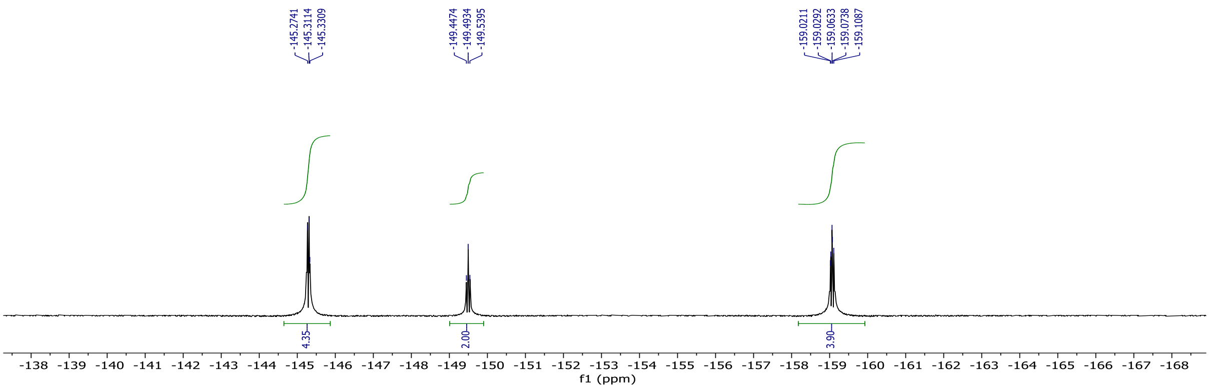

Figure S16. ^{19}F NMR Spectrum of **8** ($CDCl_3$, 298 K, 471 MHz).

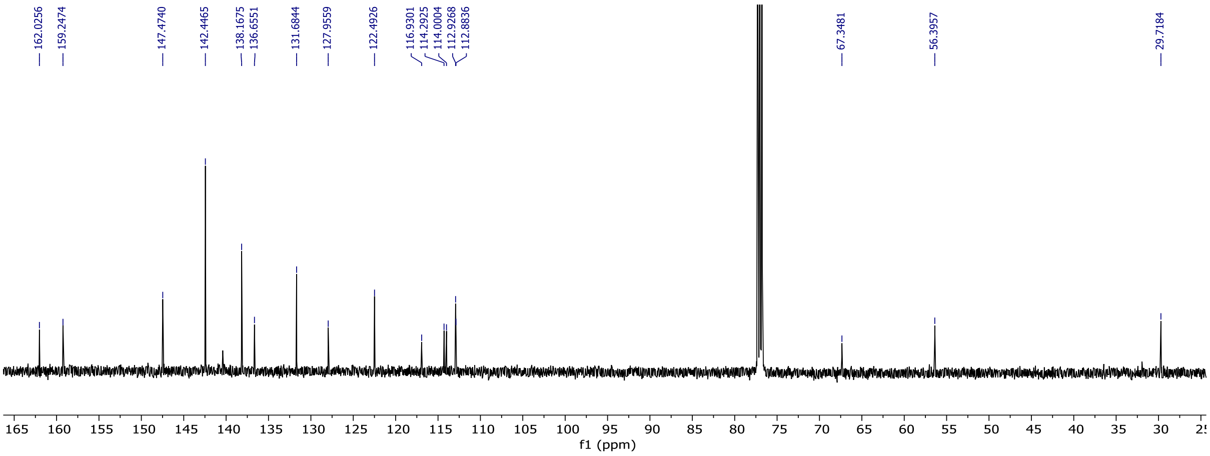

Figure S17. ^{13}C NMR Spectrum of **8** ($CDCl_3$, 298 K, 126 MHz).

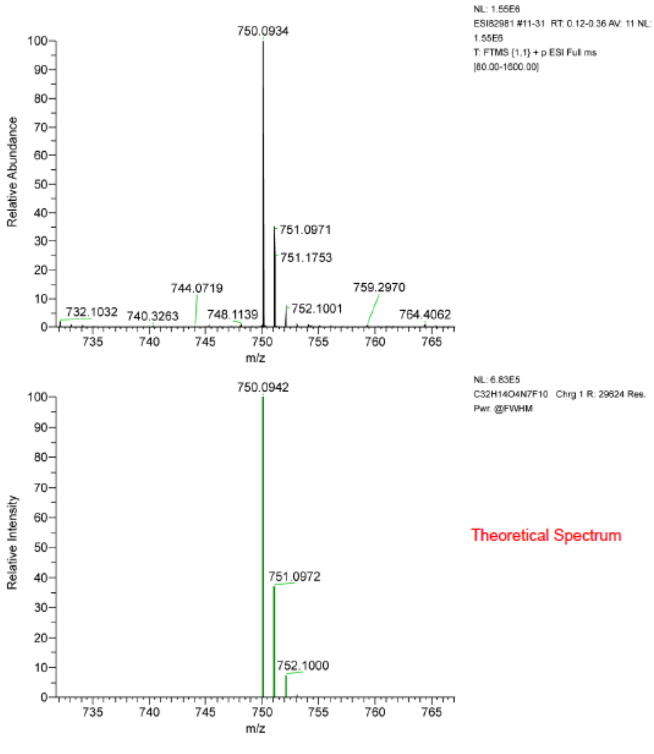

Figure S18. HRMS-ESI spectrum of compound **8**.

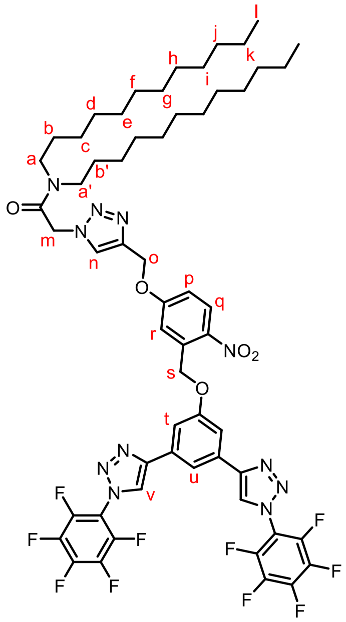

1-A. $Cu(CH_3CN)_4PF_6$ (24.5 mg, 0.0658 mmol, 0.5 eq.) and TBTA (17.5 mg, 0.0329 mmol, 0.25 eq.) were stirred in dry degassed DCM (10 mL), followed by the addition of compound **8** (98.6 mg, 0.132 mmol, 1 eq.) and 2-azido-*N*,*N*-didodecylacetamide **9** (57.5 mg, 0.132 mmol, 1 eq.). The reaction was stirred under N_2 for 48 hours. The solvent was removed in vacuo, and the residue dissolved in chloroform (20 mL). The organic mixture was washed with 10% aqueous EDTA/NH_4OH solution (2 x 10 mL), followed by brine (20 mL) and then dried over $MgSO_4$. The solvent was removed in vacuo to give the crude product. The product was purified by preparative TLC (DCM) to give a white solid (76.4 mg, 49%). ^1H NMR (500 MHz, DMSO) δ 9.29 (s, $2H_v$), 8.28 (s, $1H_n$) 8.26 (s, $1H_u$), 8.17 (s, $1H_q$), 7.70 (d, *J* = 1.4 Hz, $2H_t$), 7.50 (d, *J* = 2.8 Hz, $1H_r$), 7.33 (dd, *J* = 9.2, 2.8 Hz, $1H_p$), 5.66 (s, $2H_s$), 5.41 (s, $2H_o$), 5.35 (s, $2H_m$), 3.29 (m, $3H_a$), 3.20 (t, *J* = 7.3 Hz, $2H_{a'}$), 1.56 (t, *J* = 7.6 Hz, $2H_b$), 1.42 (p, *J* = 7.5 Hz, $3H_{b'}$), 1.28 – 1.20 (m, $36H_{c\text{-}k}$), 0.84 – 0.81 (m, $7H_l$). ^{19}F NMR (471 MHz, DMSO) δ -146.98 – -147.32 (m, 4F), -151.22 (t, *J* = 23.0 Hz, 2F) -159.44 – -161.86 (m, 4F). ^{13}C NMR (126 MHz, DMSO) δ 164.79, 162.41, 158.99, 146.49, 141.98, 141.83, 141.25, 140.11, 137.68, 135.81, 131.62, 128.04, 126.82, 125.06, 115.64, 115.23, 113.59, 112.53, 112.08, 66.74, 61.94, 50.59, 46.56, 45.61, 40.12, 40.02, 39.95, 39.86, 39.78, 39.69, 39.61, 39.52, 39.44, 39.35, 39.19, 39.02, 31.29, 29.04-28.92 (m), 28.71, 28.29, 27.09, 26.28, 26.14, 22.09, 13.91. HRMS-ESI (m/z) Calculated for $C_{58}H_{65}O_5N_{11}F_{10}$-$Na^+$: 1208.4903; found 1208.4892.

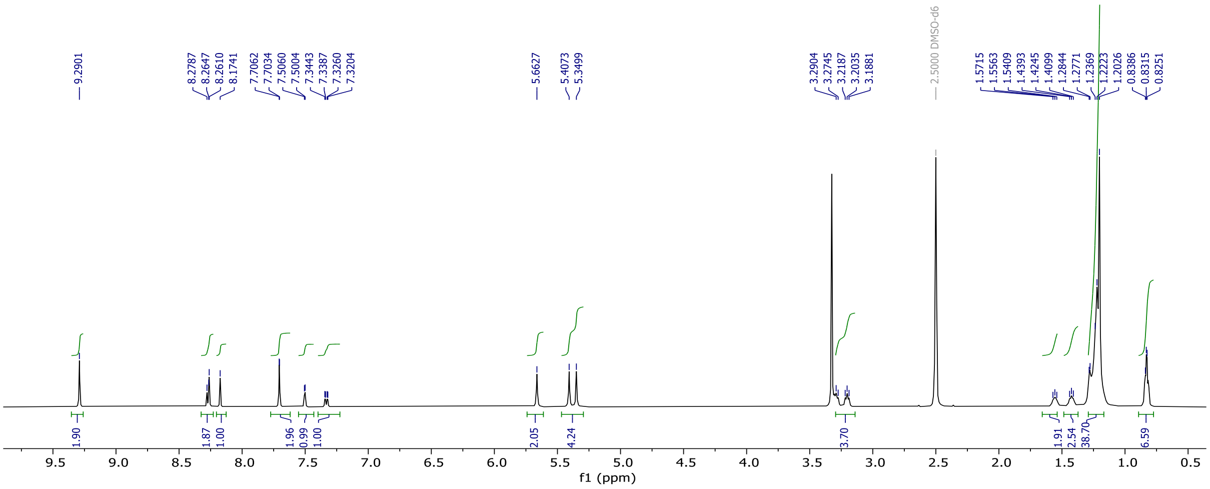

Figure S19. ^1H NMR Spectrum of **1-A** (DMSO-*d*$_6$, 298 K, 500 MHz).

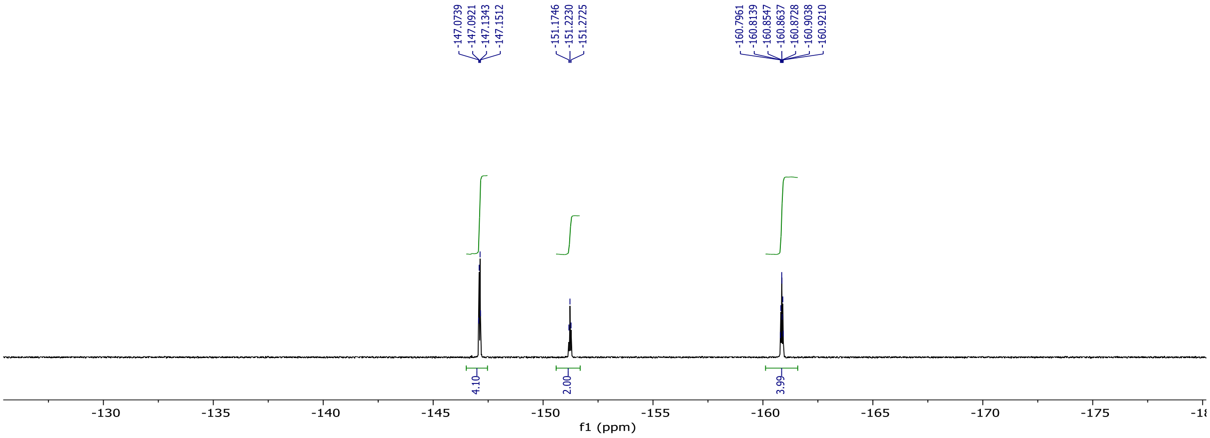

Figure S20. ^{19}F NMR Spectrum of **1-A** (DMSO-d_6, 298 K, 471 MHz).

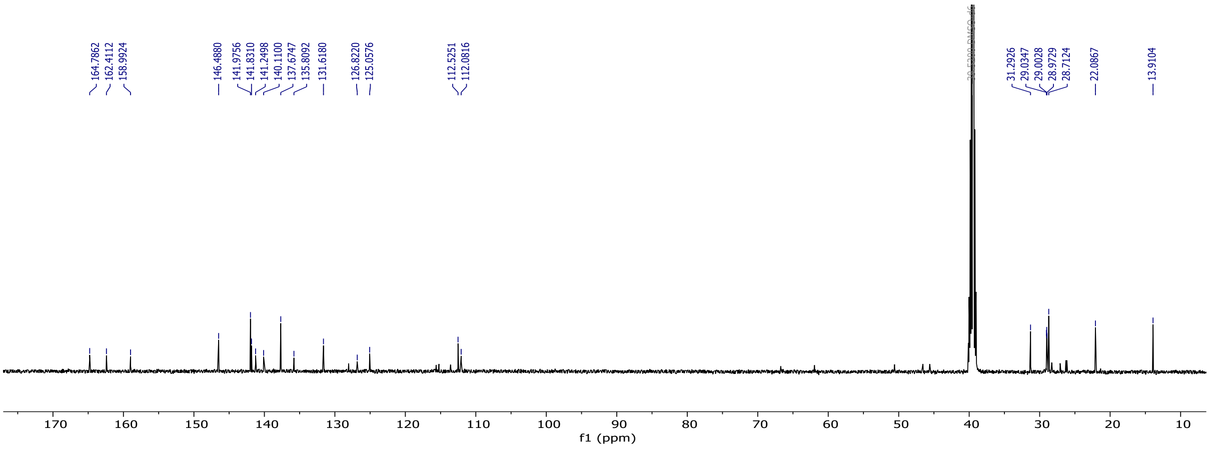

Figure S21. ^{13}C NMR Spectrum of **1-A** (DMSO-d_6, 298 K, 126 MHz).

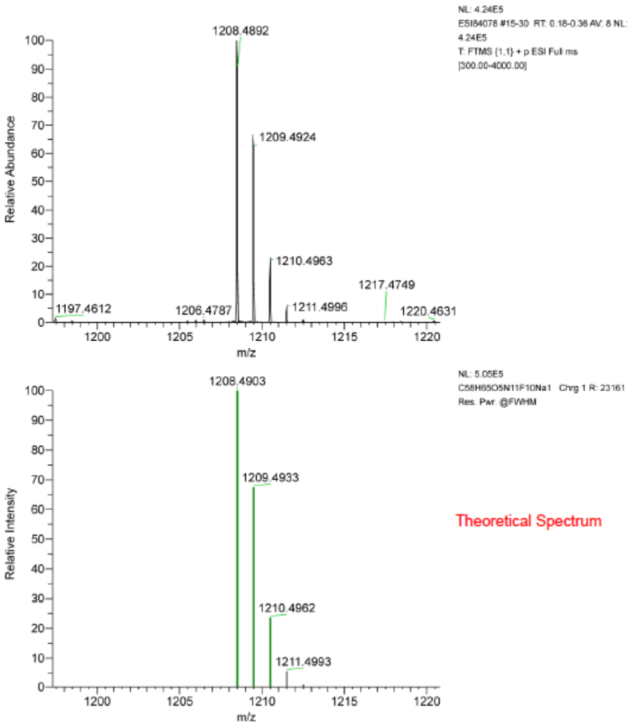

Figure S22. HRMS-ESI spectrum of compound **1-A**.

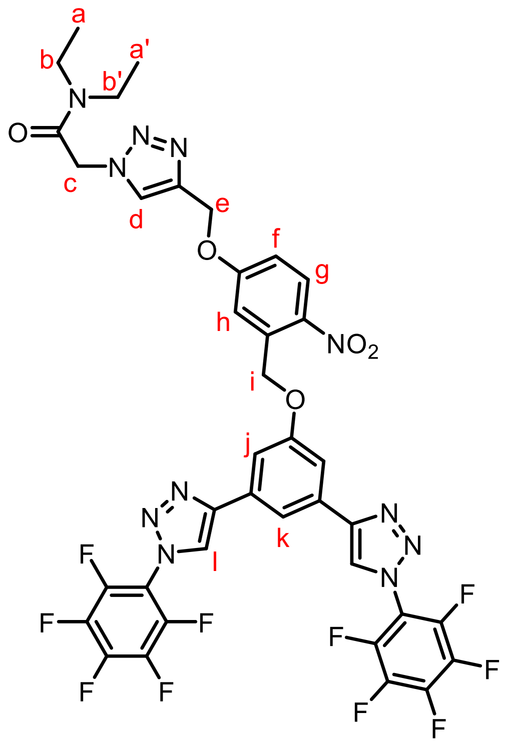

1-B. $Cu(CH_3CN)_4PF_6$ (5.86 mg, 0.0215 mmol, 0.5 eq.) and TBTA (5.69 mg, 0.0107 mmol, 0.25 eq.) were stirred in dry degassed DCM (5 mL), followed by the addition of compound **8** (32 mg, 0.0430 mmol, 1 eq.) with 2-azido-*N*,*N*-diethylacetamide **10** (14 mg, 0.0859 mmol, 2 eq.). The reaction was stirred under N_2 for 48 hours. The solvent was removed in vacuo, and the residue dissolved in chloroform (20 mL). The organic mixture was washed with 10% aqueous EDTA/NH_4OH solution (2 x 10 mL), followed by brine (20 mL) and then dried over $MgSO_4$. The solvent was removed in vacuo to give the crude product. The product was purified by preparative TLC (10% MeOH in DCM) to give a white solid (17 mg, 44%). ^{1}H NMR (500 MHz, DMSO) δ 9.29 (s, 2H_l), 8.28 (s, 1H_d), 8.26 (s, 1H_k), 8.19 (s, 1H_g), 7.71 (s, 2H_j), 7.51 (d, *J* = 2.8 Hz, 1H_h), 7.34 (dd, *J* = 9.2, 2.8 Hz, 1H_f), 5.67 (s, 2H_i), 5.43 (s, 2H_e), 5.36 (s, 2H_c), 3.39 (q, *J* = 7.1 Hz, 2H_a), 3.27 (m, 2$H_{a'}$), 1.18 (t, *J* = 7.1 Hz, 3H_b), 1.02 (t, *J* = 7.1 Hz, 3$H_{b'}$). ^{19}F NMR (471 MHz, DMSO-d_6) δ -147.11 (d, *J* = 20.4 Hz, 4F), -151.15 (t, *J* = 22.8 Hz, 2F), -160.80 (t, *J* = 21.5 Hz, 4F). ^{13}C NMR (126 MHz, DMSO-d_6) δ 164.45, 162.39, 159.03, 146.49, 141.98, 141.85, 141.26, 140.18, 137.69, 135.76, 131.62, 126.86, 125.09, 115.66, 115.33, 113.65, 112.51, 112.12, 66.79, 61.93, 50.55, 13.95, 12.83. HRMS-ESI (m/z) Calculated for $C_{38}H_{26}O_5N_{11}F_{10}$-$H^+$: 906.1953; found 906.1946.

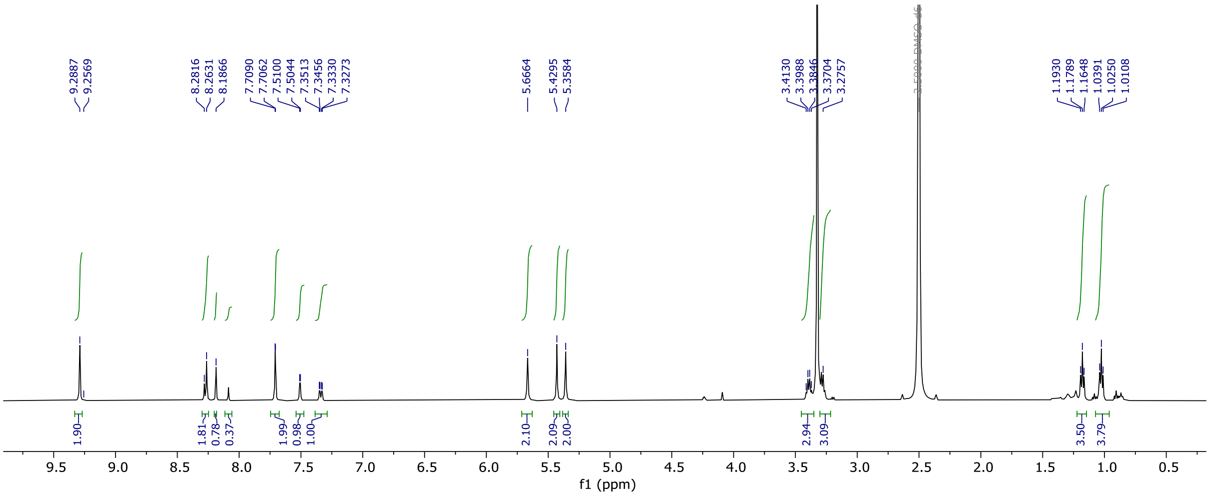

Figure S23. ^{1}H NMR Spectrum of **1-B** (DMSO-d_6, 298 K, 500 MHz).

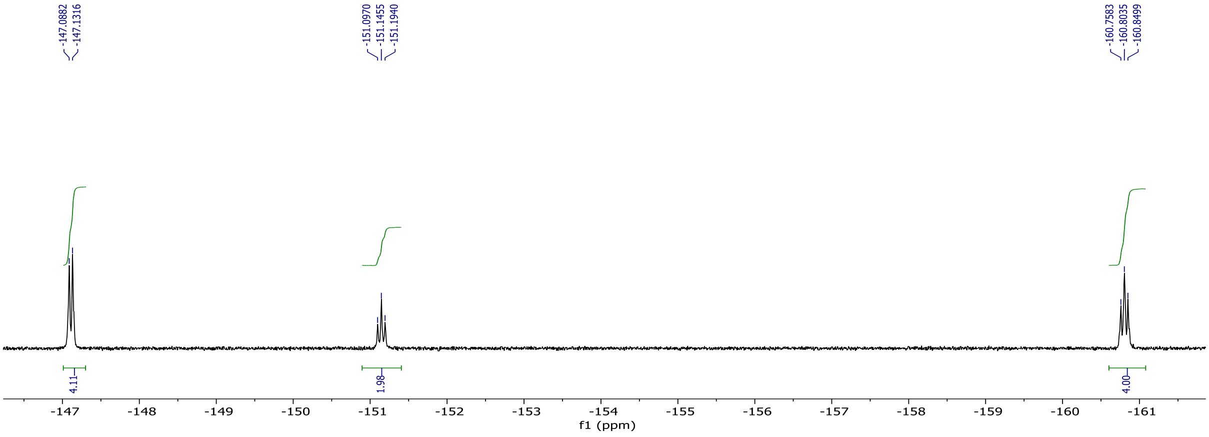

Figure S24. ^{19}F NMR Spectrum of **1-B** (DMSO-d_6, 298 K, 471 MHz).

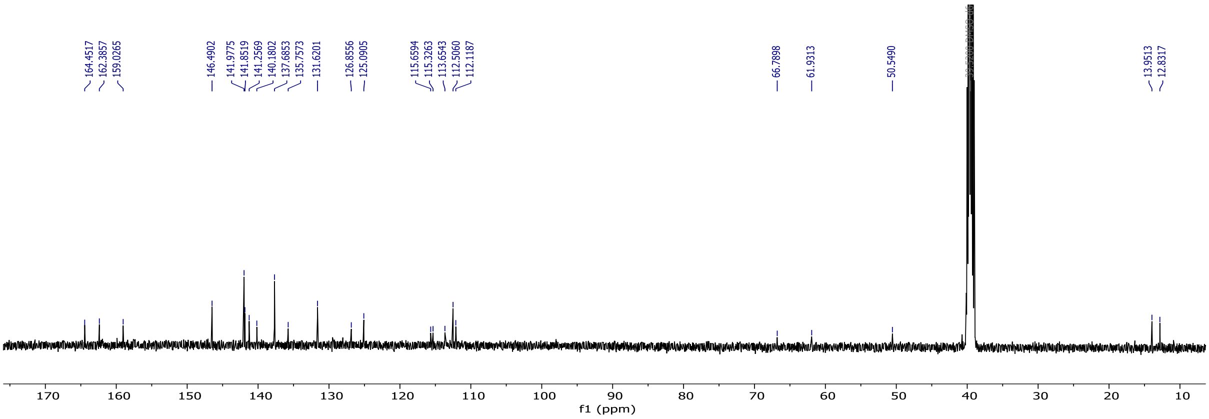

Figure S25. ^{13}C NMR Spectrum of **1-B** (DMSO-d_6, 298 K, 126 MHz).

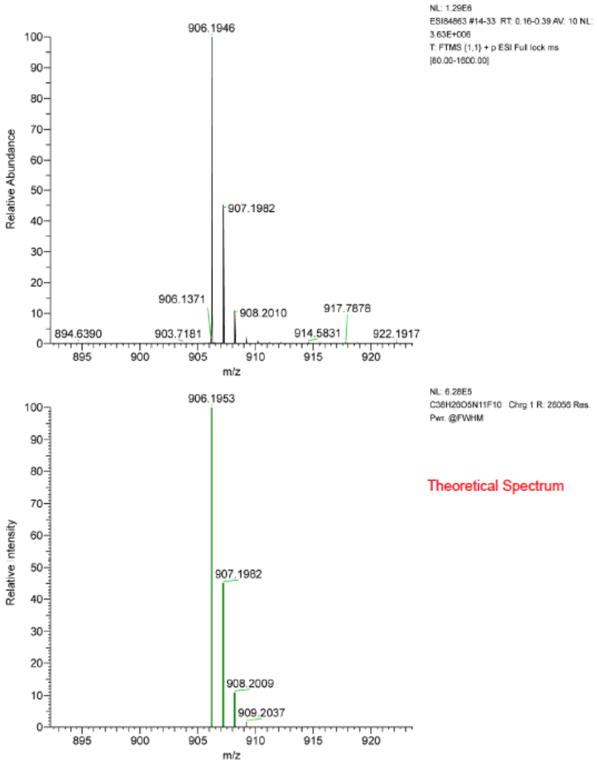

Figure S26. HRMS-ESI spectrum of compound **1-B**.

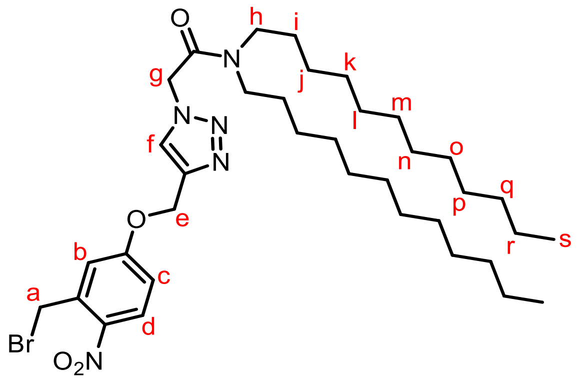

Compound 15. $Cu(CH_3CN)_4PF_6$ (252.4 mg, 0.9255 mmol, 0.5 eq.) and TBTA (245.5 mg, 0.4628 mmol, 0.25 eq.) were stirred in dry degassed DCM (15 mL), followed by the addition of compound **7** (500 mg, 1.851 mmol, 1 eq.) and 2-azido-*N*,*N*-didodecylacetamide **9** (808 mg, 1.851 mmol, 1 eq.)The reaction was stirred under N_2 for 48 hours. The solvent was removed in vacuo, and the residue dissolved in chloroform (20 mL). The organic mixture was washed with 10% aqueous EDTA/NH_4OH solution (2 x 10 mL), followed by brine (20 mL) and then dried over $MgSO_4$. The solvent was removed in vacuo to give the crude product. The product was purified by column chromatography (10% EtOAc in DCM) to give a white solid (783 mg, 60%). ^1H NMR (600 MHz, DMSO) δ 8.19 (s, $1H_f$), 8.15 (d, *J* = 9.1 Hz, $1H_d$), 7.43 (d, *J* = 2.8 Hz, $1H_b$), 7.28 (dd, *J* = 9.2, 2.8 Hz, $1H_c$), 5.42 (s, $2H_a$), 5.33 (s, $2H_e$), 4.95 (s, $2H_g$), 3.30 (m, $2H_h$), 3.23 (t, *J* = 7.6 Hz, $2H_{h'}$), 1.57 (q, *J* = 7.5 Hz, $2H_i$), 1.44 (q, *J* = 7.5 Hz, $2H_{i'}$), 1.30 – 1.23 (m, $32H_{j\text{-}r}$), 0.85 (m, $6H_s$). ^{13}C NMR (151 MHz, DMSO) δ 164.77, 161.83, 141.14, 140.57, 135.50, 128.24, 126.82, 118.61, 115.17, 61.93, 50.57, 46.57, 45.63, 31.28, 30.37, 29.02, 29.00, 28.97, 28.94, 28.91, 28.70, 28.30, 27.11, 26.29, 26.14, 22.08, 13.90. HRMS-ESI (m/z) Calculated for $C_{36}H_{61}O_4N_5Br\text{-}H^+$: 706.3901; found 706.3899.

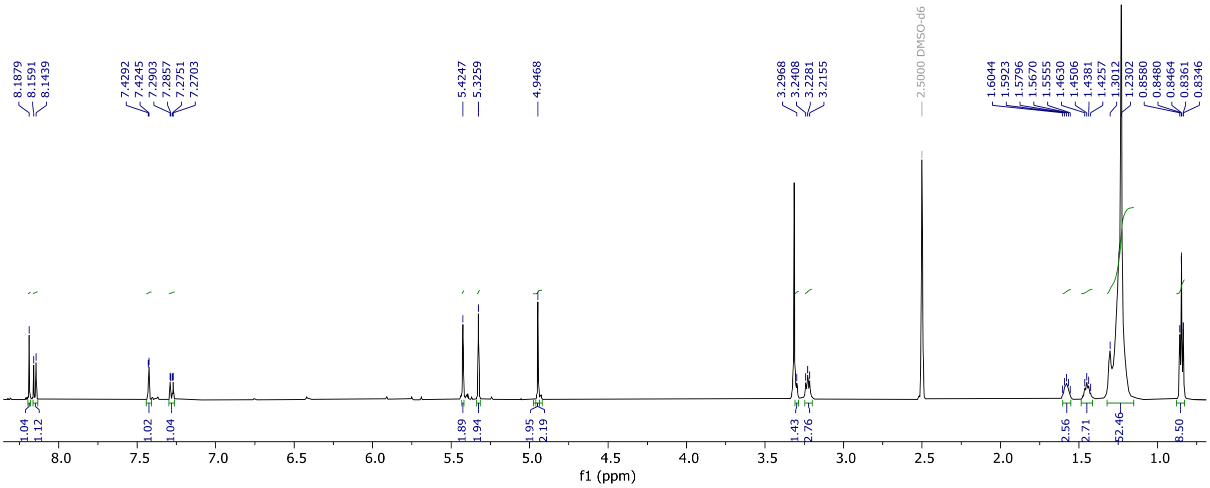

Figure S27. ^1H NMR Spectrum of **15** (DMSO-d_6, 298 K, 600 MHz).

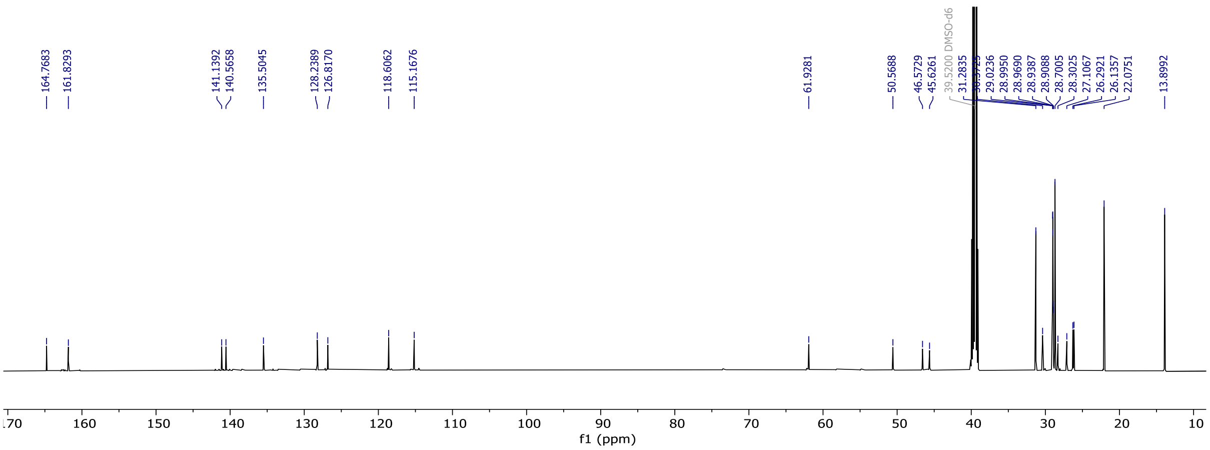

Figure S28. ^{13}C NMR Spectrum of **15** (DMSO-d_6, 298 K, 151 MHz).

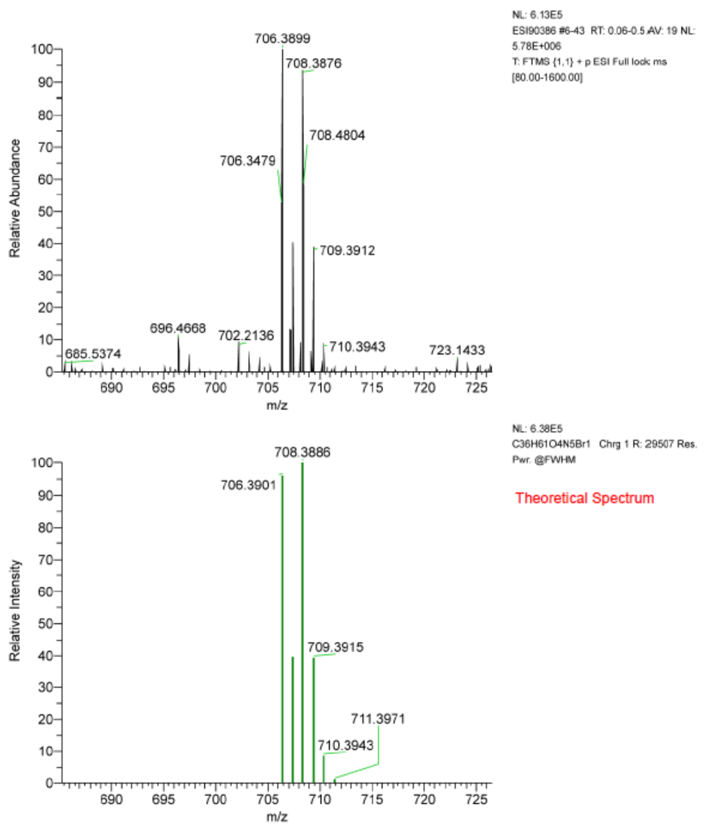

Figure S29. HRMS-ESI spectrum of compound **15**.

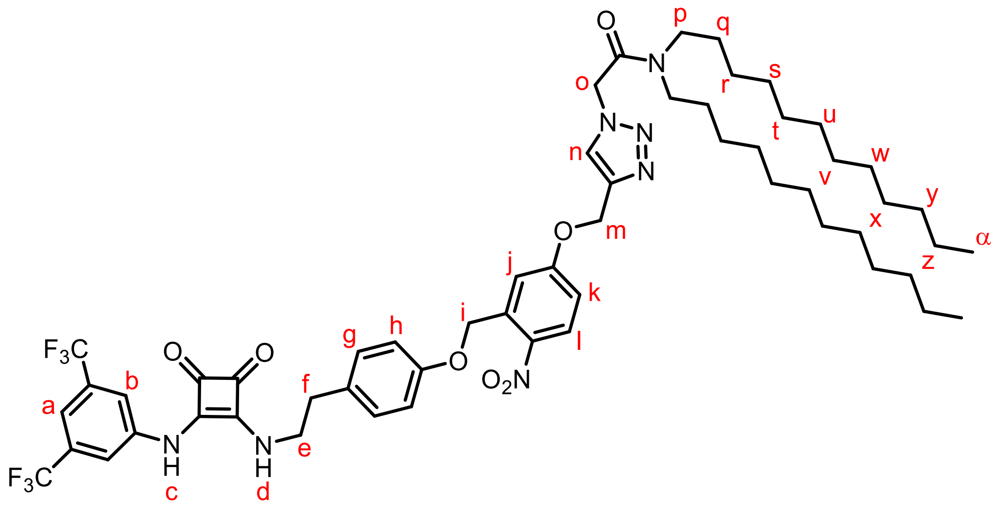

2-A. Carrier **2** (30.0 mg, 0.0674 mmol, 1 eq.) was stirred with K_2CO_3 (23.0 mg, 0.169, 2.5 eq.) in DMF (2.5 mL) at 0 °C for 10 mins. Compound **15** (48.0 mg, 0.0674 mmol, 1 eq.) was added, the reaction was warmed to 45 °C and then left to stir for 16 hrs. The solvent was removed in vacuo and the crude was suspended in EtOAc (10 mL), washed with water (2 x 10 mL) then dried over $MgSO_4$. The product was purified by preparative TLC (EtOAc and hexane 1:1) to give a pale yellow solid (15 mg, 21%). ^{1}H NMR (500 MHz, Acetone) δ 8.27 (d, J = 9.1 Hz, 1H$_l$), 8.25 (s, 1H$_n$), 8.02 (s, 1H$_a$), 7.72 (s, 1H$_c$), 7.69 (s, 2H$_b$), 7.22 (dd, J = 9.2, 2.8 Hz, 1H$_k$), 7.14 (d, J = 2.7 Hz, 1H$_j$), 7.07 (t, J = 6.2 Hz, 1H$_d$), 7.03 – 6.97 (m, 2H$_g$), 6.71 – 6.64 (m, 2H$_h$), 5.98 (s, 2H$_i$), 5.39 (s, 2H$_m$), 5.27 (s, 2H$_o$), 3.91 (q, J = 7.2 Hz, 2H$_e$), 3.44 (t, J = 7.8 Hz, 2H$_p$), 3.34 (t, J = 7.6 Hz, 2H$_{p'}$), 2.85 – 2.80 (m, 2H$_f$),1.71 (s, 2H$_q$), 1.54 (s, 2H$_{q'}$), 1.40 – 1.27 (m, 36H$_{r-z}$), 0.92 – 0.82 (m, 6H$_α$). ^{19}F NMR (471 MHz, Acetone) δ -63.24 (6F, 2 x CF_3). ^{13}C NMR (126 MHz, Acetone) δ 188.61, 184.94, 171.26, 165.53, 164.17, 163.60, 156.97, 143.48, 142.70, 141.58, 137.14, 133.11, 130.65, 129.76, 129.42, 127.29, 124.27, 121.27, 117.82, 116.19, 115.25, 113.99, 63.12, 53.15, 51.66, 48.04, 47.38, 47.04, 37.66, 32.67, 28.34, 27.66, 27.54, 23.36, 14.39. HRMS-ESI (m/z) Calculated for $C_{56}H_{73}O_7N_7F_6$-H$^+$: 1070.5548; found 1070.5535.

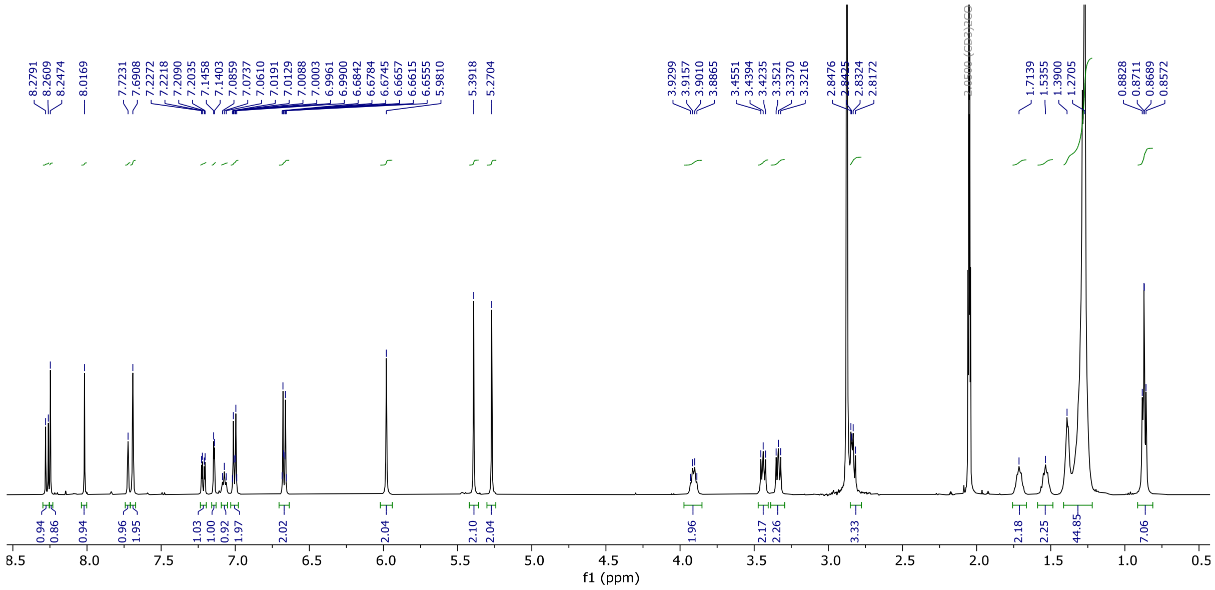

Figure S30. ^{1}H NMR Spectrum of **2-A** (Acetone-d_6, 298 K, 500 MHz).

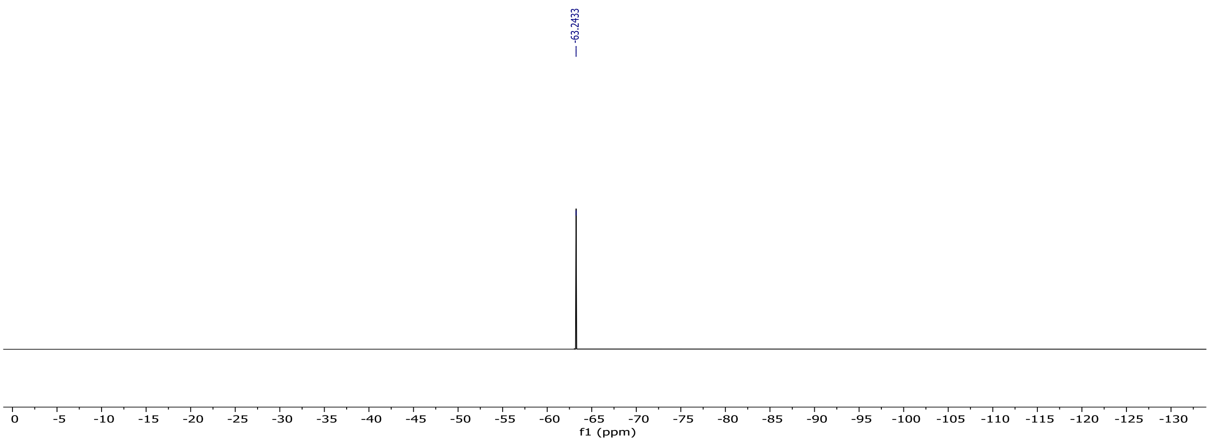

Figure S31. ^{19}F NMR Spectrum of **2-A** (Acetone-d_6, 298 K, 471 MHz).

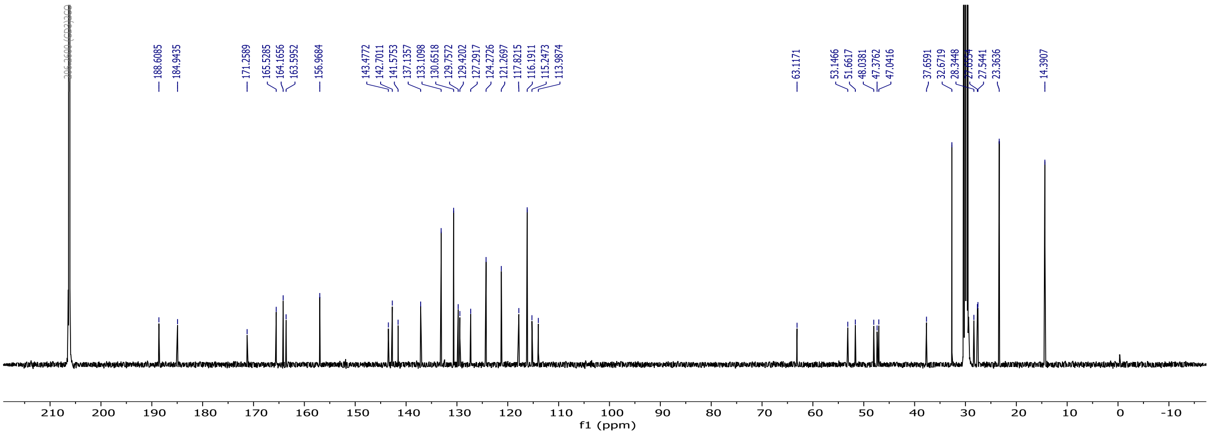

Figure S32. ^{1}H NMR Spectrum of **2-A** (Acetone-d_6, 298 K, 126 MHz).

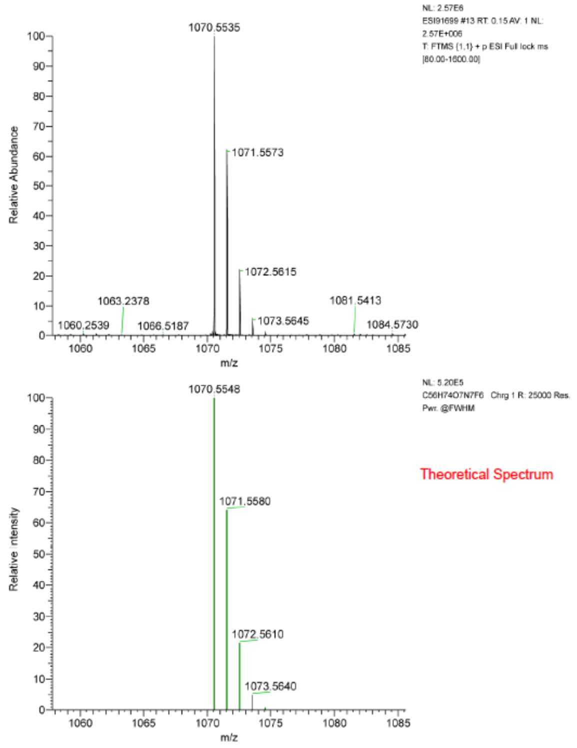

Figure S33. HRMS-ESI spectrum of compound **2-A**.

3. ^{1}H NMR Titration Experiments

Anion binding constants were determined by ^{1}H NMR titrations using a Bruker AVIII 500 spectrometer at 500 MHz and 298 K. The initial sample volumes were 0.5 mL, at a concentration of 1 mM of host dissolved in acetone-d_6 or 2.5% D_2O-acetone-d_6 (v/v). Known volumes of the anion guest, as the tetrabutylammonium (TBA) salt in the same solvent were added to the host and the spectra were recorded after each addition. The chemical shift perturbations of the host spectra were monitored as a function of guest concentration. The spectra were referenced to the residual solvent peak. The data was analysed using a global fit procedure using the Bindfit program,[9,10] using non-linear least squares analysis to obtain the best fit between observed and calculated chemical shifts for the 1:1 binding stoichiometry. The association of guest and host was fast on the NMR timescale.

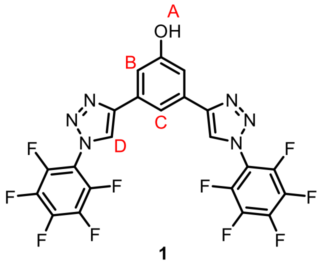

1

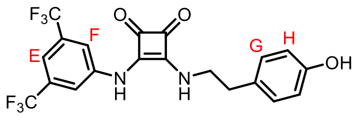

2

Figure S34. Carriers **1** and **2**.

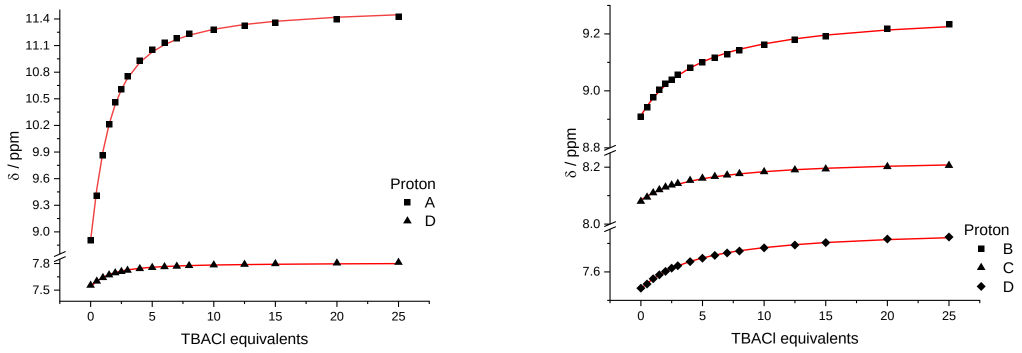

Figure S35. Chloride binding isotherms for **1** in acetone-d_6 (left) and in 2.5% D_2O-acetone-d_6 (right). Experimental data shown by symbols, fitted 1:1 binding isotherm shown by solid red lines.

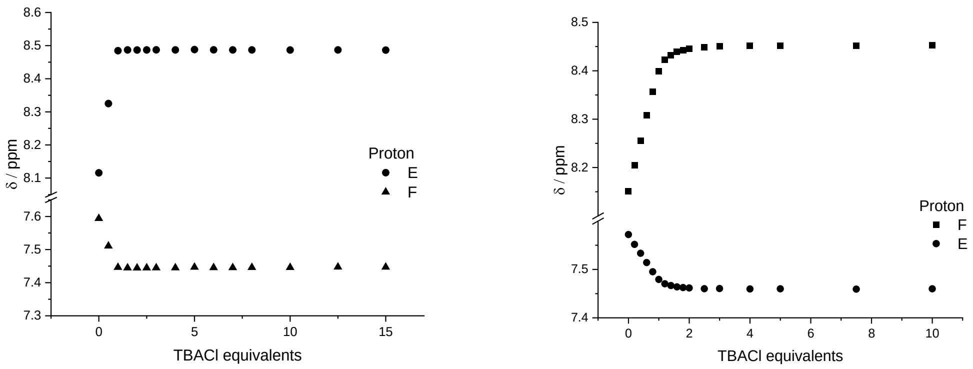

Figure S36. Chloride binding isotherms for **2** in acetone-d_6 (left) and in 2.5% D_2O-acetone-d_6 (right). Experimental data shown by symbols. In each case the binding affinity was too strong to be determined (>10^4 M^{-1}).

4. Photocleavage Experiments

A 2 mM sample of each anchored carrier in DMSO was subjected to irradiation at 365 nm using an LED (irradiation intensity 1.3 W) and followed by ^{1}H NMR after increasing irradiation time intervals.

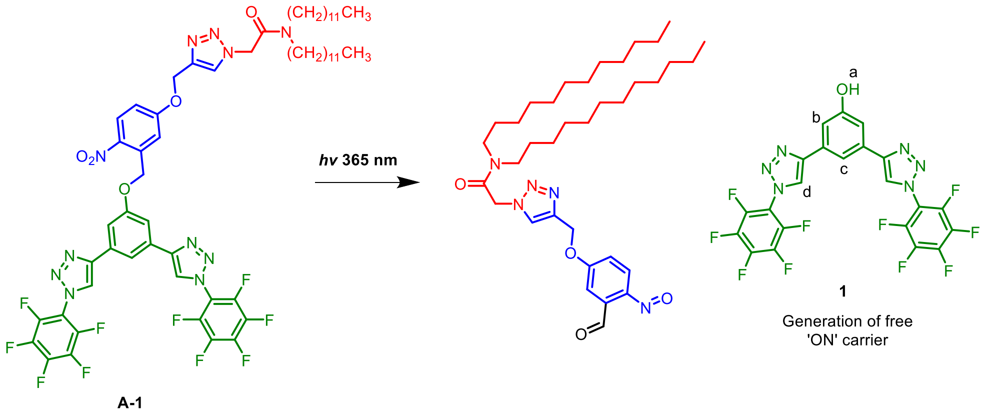

Figure S37. Generation of **1** when **A-1** is irradiated with 365 nm UV light.

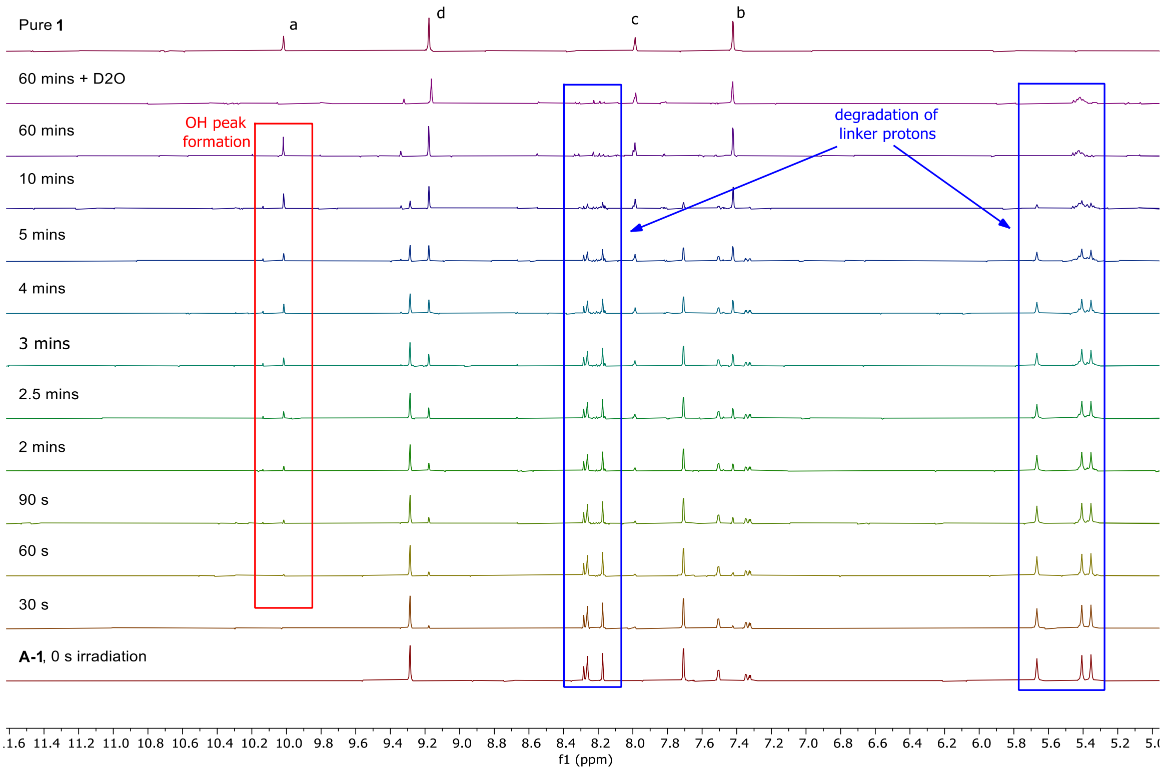

Figure S38. Photocleavage of **A-1** with 365 nm UV light (2 mM sample in d_6-DMSO) and generation of the free carrier over time from 0 to 60 mins. Membrane anchor protons not shown.

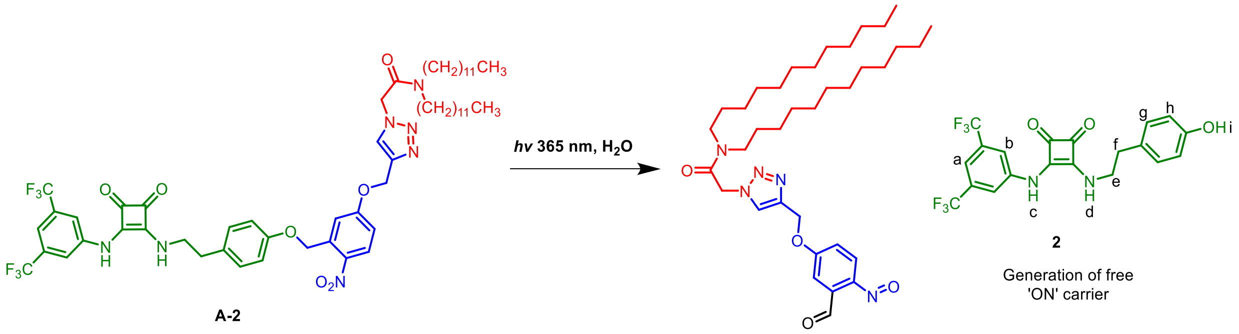

Figure S39. Generation of **2** when **A-2** is irradiated with 365 nm UV light.

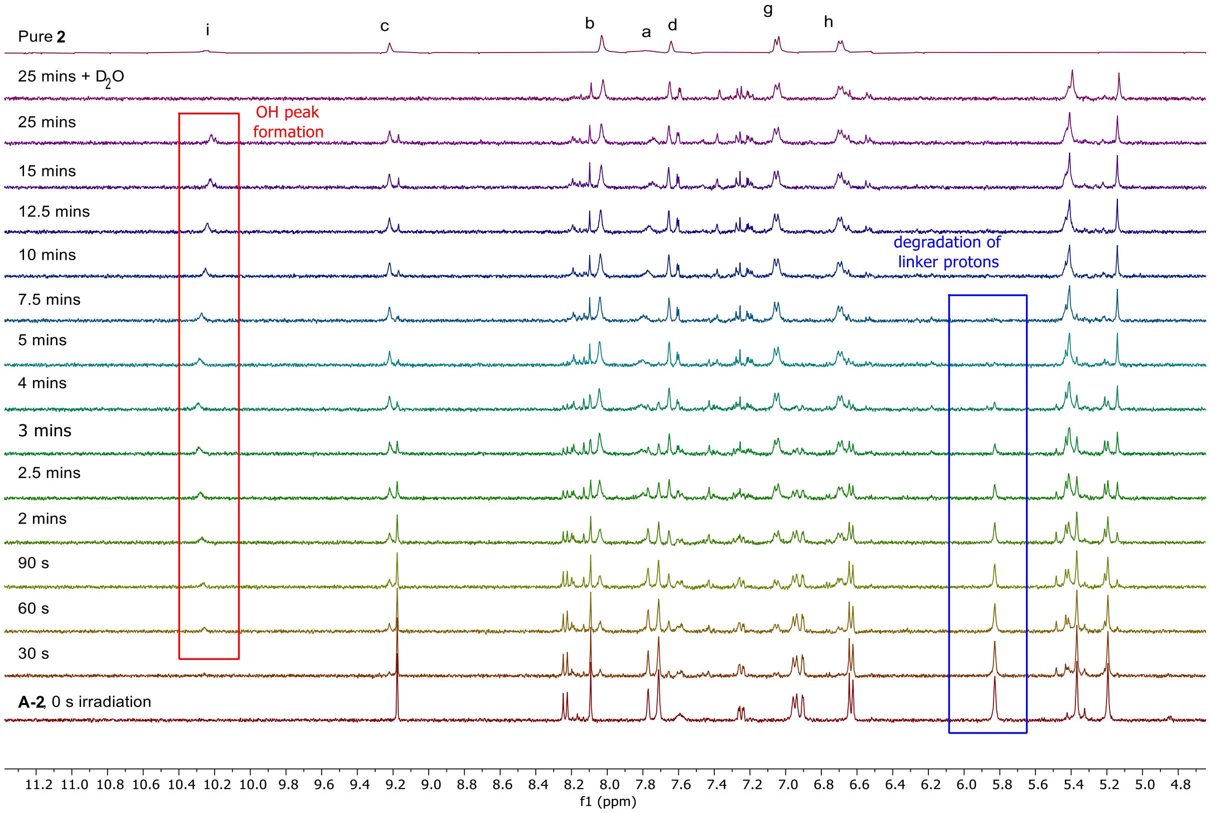

Figure S40. Photocleavage of **A-2** nitrobenzyl group with 365 nm UV light (2 mM sample in *d*-DMSO) leading to the generation of the free carrier **2** over time from 0 to 25 mins. Membrane anchor protons not shown.

The conversion to the free carrier was determined as follows:
t_1 was calculated by fitting the data to an exponential decay function (equation S1) which was then used to derive a half-life of **1-A** and **2-A** using equation S2. The results are presented in **Table S1**.

$$y = A_1 e^{\left(\frac{-x}{t_1}\right)} \qquad \text{(S1)}$$

$$t_{\frac{1}{2}} = t_1 ln(2) \qquad \text{(S2)}$$

Table S1. Calculated t_1 and $t_{½}$ resulting from the generation of **1** and **2** from **1-A** and **2-A** respectively.

	t_1 (mins)	$t_{½}$ (secs)
**1**	2.18	91
**2**	1.98	82

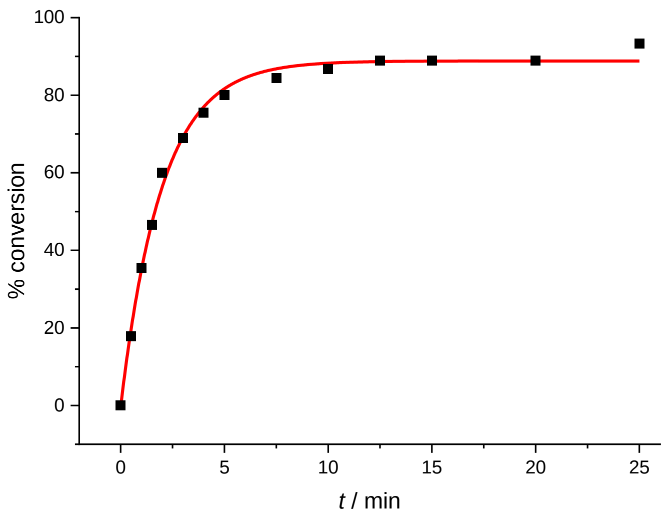

Figure S41. Generation of carrier **2** from **2-A** in DMSO solution by irradiation with 365 nm LED (1.3 W).

5. Anion Transport Experiments

Vesicle Preparation

A thin film of lipid (1-palmitoyl-2-oleoyl-sn-3-phosphatidylcholine (POPC) or dipalmitoyl phosphatidylcholine (DPPC)) was formed by evaporating a chloroform solution of the lipid under reduced pressure on a rotary evaporator (40 °C) and then under high vacuum for 6 hours. Then the lipid film was hydrated by vortexing with 1 mL of the prepared internal buffer solution. The lipid suspension was then subjected to 5 freeze-thaw cycles using liquid nitrogen and a water bath (40°C) followed by extrusion 19 times through a polycarbonate membrane (pore size 200 nm). Extrusion was performed at 50°C in the case of DPPC lipids. Extra-vesiclular components were removed by size exclusion chromatography on a Sephadex G-25 column with 100 mM NaCl, 10 mM HEPES, pH 7.0. Final conditions: LUVs (2.5 mM lipid); inside 100 mM NaCl, 10 mM HEPES, 1 mM HPTS, pH 7.0; outside: 100 mM NaCl, 10 mM HEPES, pH 7.0. Vesicles for the sodium gluconate assay were prepared by the same procedure, substituting NaCl for NaGluconate in the buffer solution

The HPTS Assay[11,12]

In a typical experiment, the LUVs containing HPTS (25 µL, final lipid concentration 31 µM) were added to buffer (1950 µL of 100 mM NaCl, 10 mM HEPES, pH 7.0) at 25°C under gentle stirring. A pulse of NaOH (20 µL, 0.5 M) was added at 40 secs to initiate the experiment. At 100 s the test transporter (various concentrations, typically in 5 µL DMSO) was added, followed by detergent (25 µL of Triton X-100 in 7:1 (v/v) H_2O-DMSO) at 300 secs to lyse the vesicles for calibration. The fluorescence emission was monitored at λ_{em} = 510 nm (λ_{ex} = 460/405 nm). For each compound, Hill plots were fitted to at-least 7, and up to 10 data points spanning the required concentration range, and each individual concentration was repeated at-least twice and averaged.

The fractional fluorescence intensity (I_{rel}) was calculated using Equation S3, where R_t is the fluorescence ratio at time t, R_0 is the fluorescence ratio at time 0, and R_d is the fluorescence ratio after the addition of detergent.

$$I_{rel} = \frac{R_t - R_0}{R_d - R_0} \qquad \text{(S3)}$$

The fractional fluorescence intensity (I_{rel}) at 288 s just prior to lysis, defined as the fractional activity y, was plotted as a function of the ionophore concentration (x / µM). Hill coefficients (n) and EC_{50} values were calculated by fitting to the Hill equation (S4)

$$y = y_0 + (y_{max} - y_0) \cdot \frac{x^n}{EC50^n + x^n} \qquad \text{(S4)}$$

where y_0 is the fractional activity in the absence of transporter, y_{max} is the fractional activity in with excess transporter, x is the transporter concentration in the cuvette. Hill plots were fitted to at-least 8 data points spanning the required concentration range.

HPTS assay following photo-irradiation

The HPTS assay was modified to allow for *in-situ* irradiation of the anchored carriers, and carried out as follows: the desired concentration of the anchored carriers in DMSO (5 µL) are added to the lipid suspension (POPC LUVs containing 1 mM HPTS, 100 mM internal and external NaCl, buffered with 10 mM HEPES at pH 7.0) and stirred for 1 minute to allow for incorporation into the bilayer. The sample was then irradiated with 365 nm UV light using an LED irradiation intensity (~1.3 W). The transport assay was initiated with a base pulse of NaOH (20 µL, 0.5 M) and the change in ratiometric emission (λ_{em} = 510 nm; λ_{ex} = 460/405 nm) was monitored over time, before calibration with Triton-X100.

Normalisation procedure. Under the standard HPTS assay condition (in which the base pulse is added prior to the transporter), the initial instantaneous jump in fluorescence intensity observed upon addition of NaOH (due to external HPTS absorbed to the vesicle surface, reporting on the increase in external pH) occurs prior to the carrier addition, and is removed by normalisation. Under the experimental conditions used here for the *in-situ* irradiation conditions (in which the carrier is added to the membrane prior to the base pulse), this instantaneous jump happens simultaneously with transport. For normalisation, the intensity recorded immediately after the addition of the base pulse is therefore subtracted, according to the Equation S3, where R_0 is the fluorescence ratio immediately after the base pulse addition (Figure S42). The subsequent increase in I_{rel} reports on the increase in pH inside the vesicles due to carrier mediated transport. Hill analysis was performed as usual with the I_{rel} value immediately prior to vesicle lysis.

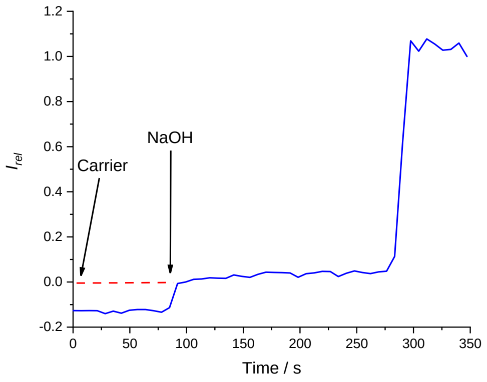

Figure S42. Normalisation procedure for in-situ photo-irradiation experiments. Data shows the change in fractional fluorescence intensity (I_{rel}) upon addition of **1-A** (0.05 µM) in DMSO to POPC LUVs. **1-A** was added to the cuvette in 5 µL DMSO, and stirred for 1 min, before the cuvette was irradiated with 365 nm light for 3 minutes. A NaOH base pulse (0.5 M) was added at t = 90 s. The data is normalised by subtraction of the I_{rel} value immediately after the addition of the base pulse.

HPTS Assay Data for compounds 1, 2, 1-A, 1-B and **2-B.**

Each plot show: Left - Change in ratiometric emission in the HPTS assay. Right: dependence on fractional activities (*y*, the relative intensity immediately prior to lysis) on concentration of transporter (black squares), and where activity allows, fit to the Hill equation (red line)

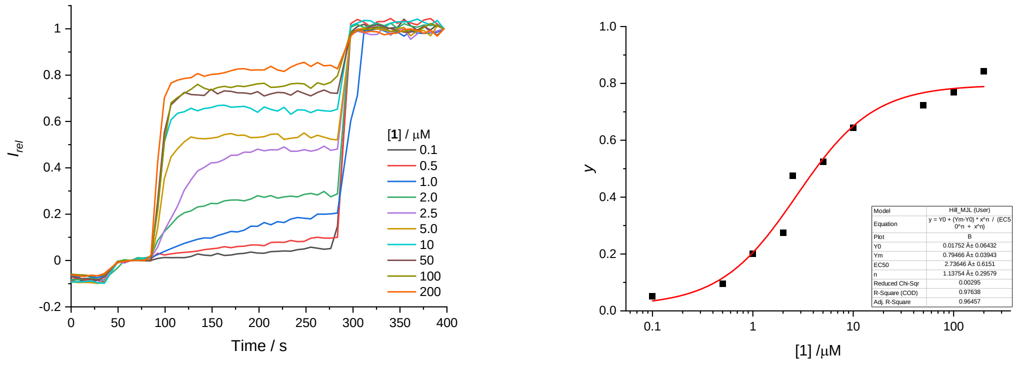

Figure S43. HPTS assay data for **1**.

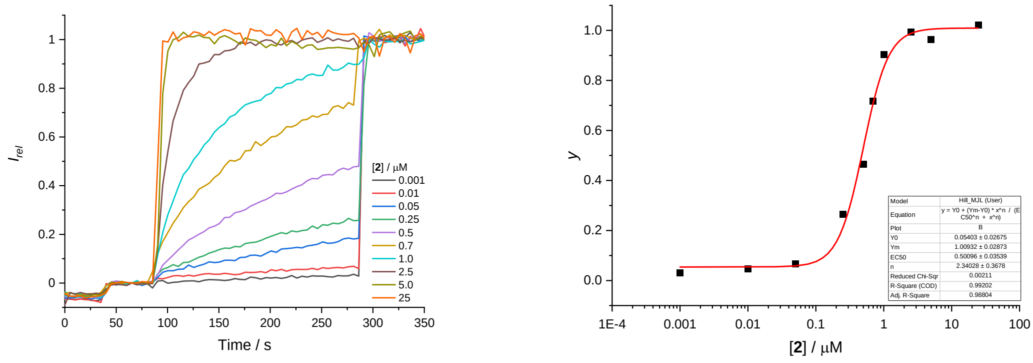

Figure S44. HPTS assay data for **2**.

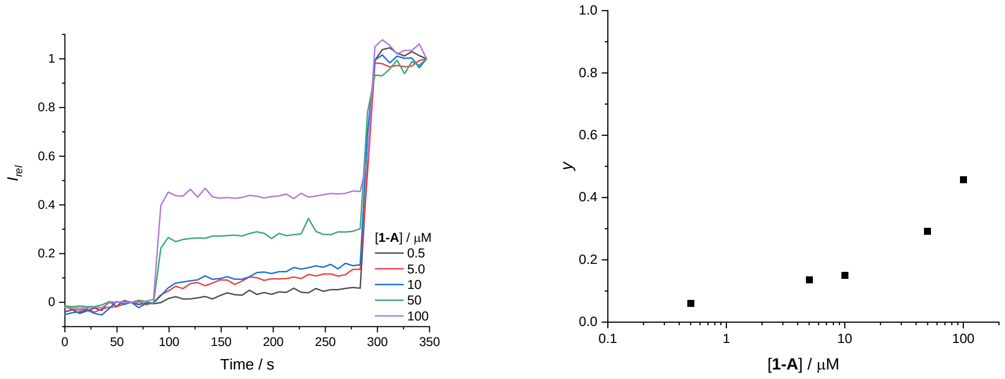

Figure S45. HPTS assay data for **1-A**.

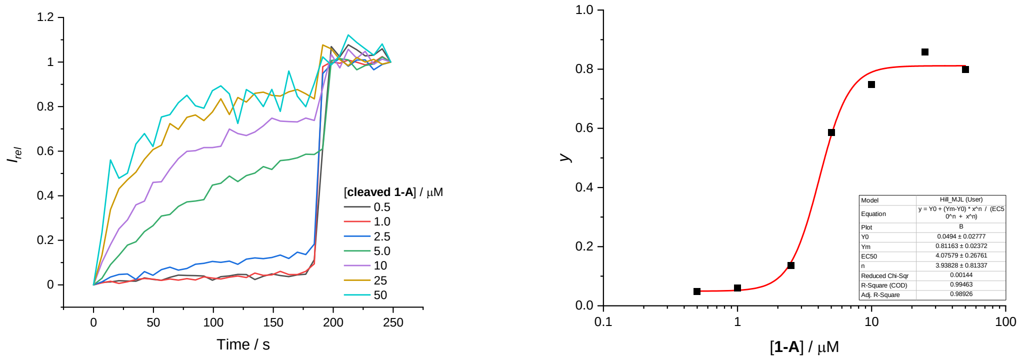

Figure S46. HPTS assay data for **1-A** after 3 mins of in situ photo-irradiation.

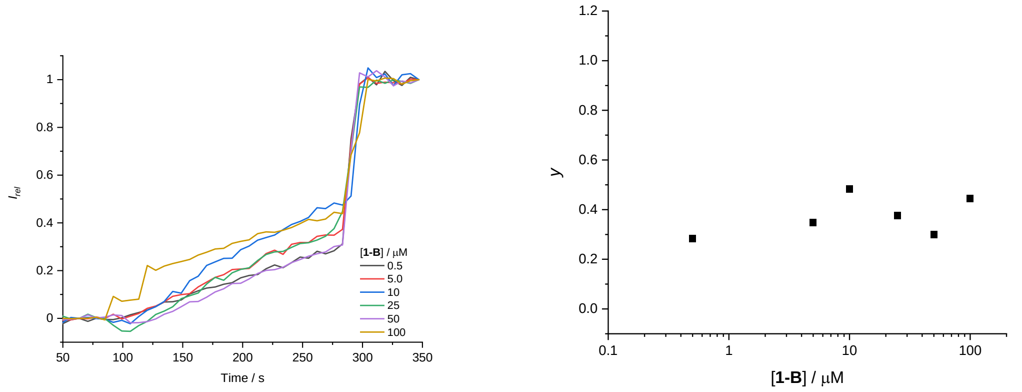

Figure S47. HPTS assay data for **1-B**.

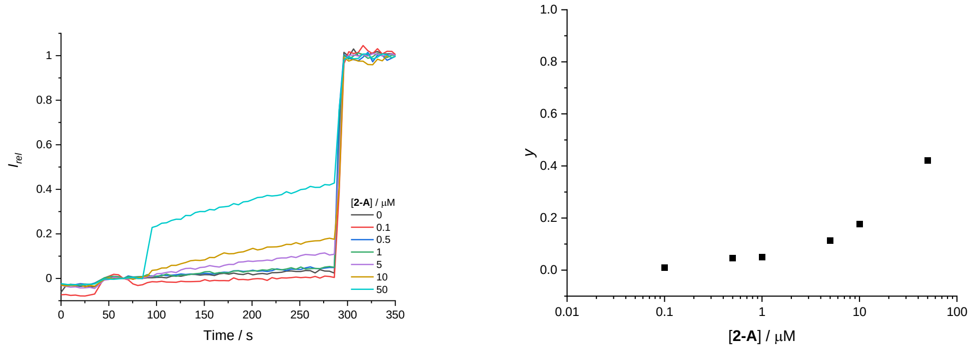

Figure S48. HPTS assay data for **2-A**.

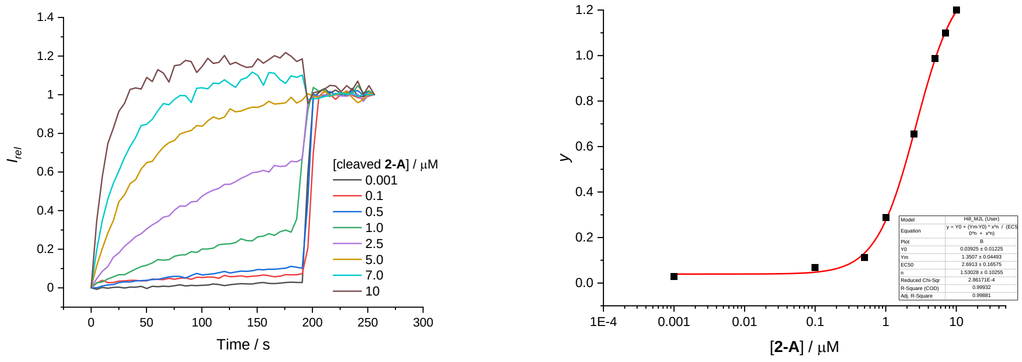

Figure S49. HPTS assay data for **2-A** after 3 mins of in situ photo-irradiation.

Sodium Gluconate HPTS Assay

POPC LUVs were prepared with an internal solution of 100 mM sodium gluconate, 10 mM HEPES, 1 mM HPTS buffered to pH 7.0 and an external buffer of 100 mM sodium gluconate, 10 mM HEPES, pH 7.0. This assay was performed at concentration of 5 µM carrier and repeated at least twice.

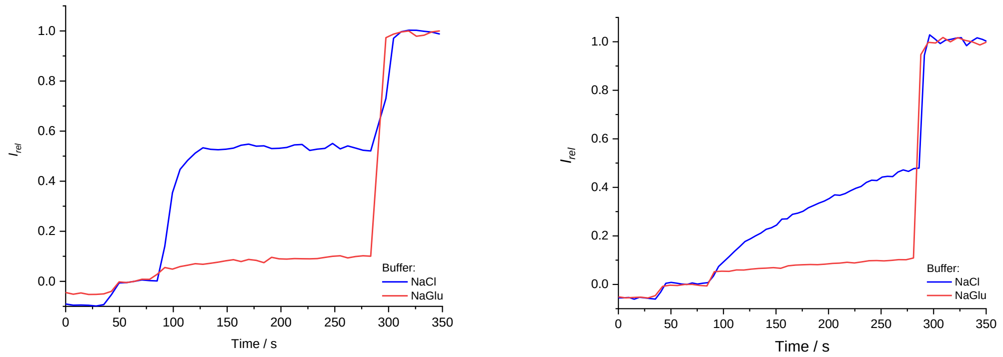

Figure S50. Gluconate assay data for: left, **1** (5 µM) and right, **2** (5 µM).

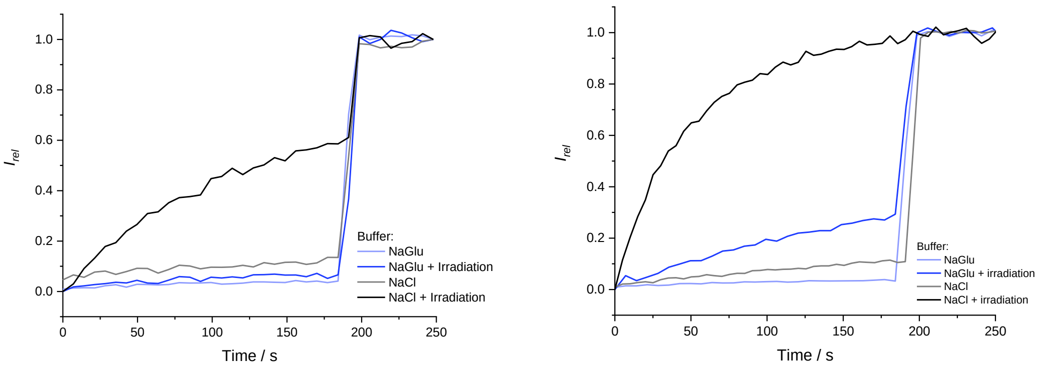

Figure S51. Gluconate assay data for: left, **1-A** (5 µM) and right, **1-B** (5 µM) before and after 3 mins of in situ photo-irradiation.

Carboxyfluorescein Leakage Assay[13]

The self-quenching dye 5(6)-Carboxyfluorescein (CF) was loaded into POPC LUVs to investigate the effect of the transporter upon vesicle integrity. POPC LUVs were prepared with an internal solution of 10 mM NaCl, 10 mM HEPES and 50 mM CF buffered to pH 7.0 and external buffer of 107 mM NaCl, 10 mM HEPES, pH 7.0. Each transport experiment was carried out as follows: the CF-containing POPC vesicles were suspended in the external buffer (1995 µL, LUV concentration 31 µM) at 25 °C and gently stirred. At 50 s, the test transporter (in 5 µL DMSO) was added. The assay was calibrated at 250 s with Triton X-100 detergent (40 µL, 7:1 (v/v) H_2O-DMSO). The time-dependent change in fluorescence intensity (λ_{ex} = 492 nm, λ_{em} = 517 nm) was monitored, and normalised according to Equation S5

$$I_{rel} = \frac{I_t - I_0}{I_{max} - I_0} \qquad \text{(S5)}$$

where I_0 = I_t before transporter addition, I_{max} = I_t after lysis.

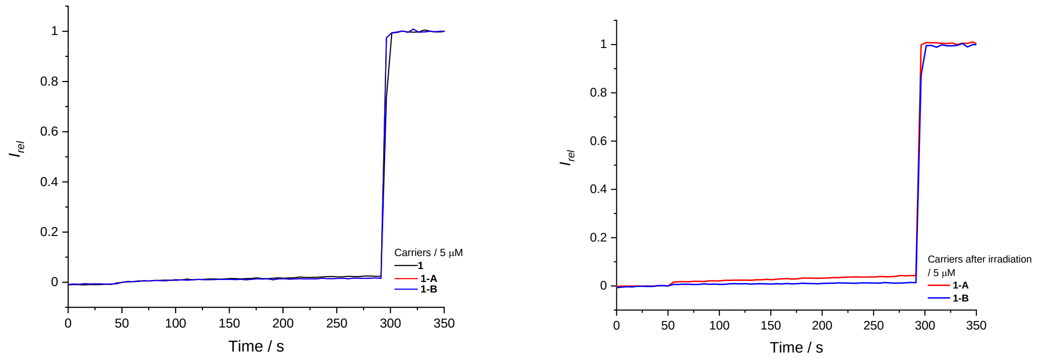

Figure S52. Left, CF assay data for **1**, **1-A** and **1-B** prior to photo-irradiation**.** Right, CF assay data for **1-A** and **1-B** after (right) 3 mins of *in situ* photo-irradiation.

HPTS Assay with DPPC lipids

The HPTS assay was carried out using the same procedure as described above, however, the POPC LUVs were replaced with dipalmitoyl phosphatidylcholine (DPPC) LUVs. These lipids are extruded at 45 °C.

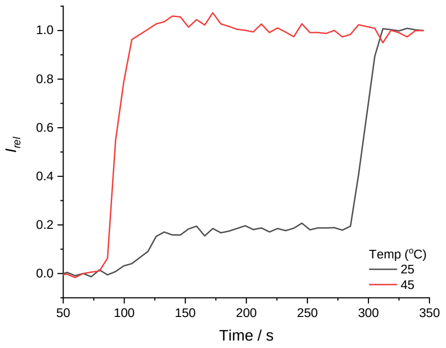

Figure S53. DPPC assay data for **1** (10 µM).

6. References

1 C. J. Brassard, X. Zhang, C. R. Brewer, P. Liu, R. J. Clark and L. Zhu, *J. Org. Chem.*, 2016, **81**, 12091–12105.

2 Y. Pan, M. Lu, Z. Peng and J. S. Melinger, *J. Org. Chem.*, 2003, **68**, 6952–6958.

3 L. M. Jin, X. Xu, H. Lu, X. Cui, L. Wojtas and X. P. Zhang, *Angew. Chem. Int. Ed.*, 2013, **52**, 5309–5313.

4 L. Li, X. X. Deng, Z. L. Li, F. S. Du and Z. C. Li, *Macromolecules*, 2014, **47**, 4660–4667.

5 N. Kalva, S. Uthaman, R. Augustine, S. H. Jeon, K. M. Huh, I. K. Park and I. Kim, *Macromol. Biosci.*, 2020, **20**, 1–12.

6 J. Pancholi, D. J. Hodson, K. Jobe, G. A. Rutter, S. M. Goldup and M. Watkinson, *Chem. Sci.*, 2014, **5**, 3528–3535.

7 M. C. Joshi, K. J. Wicht, D. Taylor, R. Hunter, P. J. Smith and T. J. Egan, *Eur. J. Med. Chem.*, 2013, **69**, 338–347.

8 H. Konishi, T. Y. Lam, J. P. Malerich and V. H. Rawal, *Org. Lett.*, 2010, **12**, 2028–2031.

9 BindFit v0.5 | Supramolecular, http://app.supramolecular.org/bindfit/.

10 D. Brynn Hibbert and P. Thordarson, *Chem. Commun.*, 2016, **52**, 12792–12805.

11 A. V. Jentzsch, D. Emery, J. Mareda, S. K. Nayak, P. Metrangolo, G. Resnati, N. Sakai and S. Matile, *Nat. Commun.*, 2012, **3**, 905.

12 N. Busschaert, R. B. P. Elmes, D. D. Czech, X. Wu, I. L. Kirby, E. M. Peck, K. D. Hendzel, S. K. Shaw, B. Chan, B. D. Smith, K. A. Jolliffe and P. A. Gale, *Chem. Sci.*, 2014, **5**, 3617–3626.

13 R. E. Dawson, A. Hennig, D. P. Weimann, D. Emery, V. Ravikumar, J. Montenegro, T. Takeuchi, S. Gabutti, M. Mayor, J. Mareda, C. A. Schalley and S. Matile, *Nat. Chem.*, 2010, **2**, 533–538.
